# A Review of Recent Developments in Driver Drowsiness Detection Systems

**DOI:** 10.3390/s22052069

**Published:** 2022-03-07

**Authors:** Yaman Albadawi, Maen Takruri, Mohammed Awad

**Affiliations:** 1 Department of Computer Science and Engineering, American University of Ras Al Khaimah, Ras Al Khaimah 72603, United Arab Emirates; yaman.albadawi@aurak.ac.ae (Y.A.); mohammed.awad@aurak.ac.ae (M.A.); 2 Department of Electrical, Electronics and Communications Engineering, American University of Ras Al Khaimah, Ras Al Khaimah 72603, United Arab Emirates

**Keywords:** biological-based measures, driver drowsiness detection, hybrid-based measures, image-based measures, vehicle-based measures

## Abstract

Continuous advancements in computing technology and artificial intelligence in the past decade have led to improvements in driver monitoring systems. Numerous experimental studies have collected real driver drowsiness data and applied various artificial intelligence algorithms and feature combinations with the goal of significantly enhancing the performance of these systems in real-time. This paper presents an up-to-date review of the driver drowsiness detection systems implemented over the last decade. The paper illustrates and reviews recent systems using different measures to track and detect drowsiness. Each system falls under one of four possible categories, based on the information used. Each system presented in this paper is associated with a detailed description of the features, classification algorithms, and used datasets. In addition, an evaluation of these systems is presented, in terms of the final classification accuracy, sensitivity, and precision. Furthermore, the paper highlights the recent challenges in the area of driver drowsiness detection, discusses the practicality and reliability of each of the four system types, and presents some of the future trends in the field.

## 1. Introduction

Based on 2017 police and hospital reports, the National Highway Traffic Safety Administration (NHTSA) identified 91,000 car accidents as being caused by drowsy drivers. These accidents resulted in 50,000 injuries. In 2019, 697 fatalities involved a drowsy driver. However, NHTSA admits that it is hard to determine the precise number of drowsy-driving accidents, injuries, or deaths and that the reported numbers are underestimates [1]. For example, a study by the American Automobile Association’s foundation for traffic safety estimated that more than 320,000 drowsy driving accidents happen each year, including 6400 fatal crashes [2]. The high numbers indicate that drowsy driving is a serious concern that needs to be addressed to mitigate its impact.

Drowsiness refers to sleepiness, often in inappropriate situations [3]. Although the state of drowsiness may only last for a few minutes, its consequences can be disastrous. The reason for entering such a state is usually attributed to fatigue, which diminishes attention and alertness levels [4]. Drowsiness may happen either by driving for long distances without enough sleep or driving at a time when the driver would typically be asleep [5]. In such cases, the main problem is the drowsy driver’s lack of concentration, resulting in a delayed response to any event on the road [6].

Fortunately, it is possible to detect driver drowsiness in its early stages and alarm the driver to avoid any potential accident. Drowsy drivers exhibit various signs, which include repeated yawning, frequent eye closure, and repeatedly departing street lanes [6]. In fact, driver drowsiness detection (DDD) techniques have been researched intensively in recent years [7,8,9,10,11,12,13]. Researchers have proposed various measures to detect these drowsiness signs as early as possible, in order to avoid accidents. These measures can be divided into four main categories: firstly, image-based measures that are obtained using a camera to analyze the driver’s movements and facial expressions; secondly, biological-based measures that relate to the driver’s bio-signals and can be recorded by placing special sensors on the driver’s body; thirdly, vehicle-based measures, which depend on monitoring the behavior and movement of the vehicle; finally, hybrid-based measures, using two or more measures. According to the literature, in 2019, Ramzan et al. [9] presented a comprehensive analysis for the existing DDD methods, as well as a detailed analysis for the commonly used classification techniques in this sector. Ramzan et al. classified the DDD techniques into three categories: behavioral, physiological, and vehicular parameter-based techniques. Then, they reviewed the top supervised learning techniques used in detecting drowsiness. In the end, they discussed the pros and cons of the three DDD in a comparative study. On the other hand, Sikander and Anwar [10] presented an in-depth review of the recent advancements in the field of driver fatigue detection. In this review, the DDD methods were categorized into five groups, depending on the extracted fatigue features, including physical features, vehicular features, biological features, subjective reporting, and hybrid features. Furthermore, the fatigue effect on driving performance was discussed, along with the existing commercial products for fatigue detection available on the market. Additionally, Dong et al. presented a review of driver inattention monitoring technologies. Inattention consists of distraction and fatigue [12]. Dong et al. summarized the detection measure into five groups, similar to Sikander and Anwar’s work [10]. In their review, Dong et al. introduced the concept of driver inattention and its effect on driving performance. Additionally, they covered some of the commercial products related to inattention detection, along with a detailed review of previous research on inattention detection.

This review contributes to the literature by covering the recently implemented DDD systems, especially those published over the past three years. Our paper classifies these systems into four categories, based on the measures used to determine the state of drowsiness. From our perspective, these measures can be image-, biological-, vehicle-, or hybrid-based.

Moreover, the review lists and tabulates the used parameters, sensors, extracted features, methods and classifiers, and quality metrics (including accuracy, sensitivity, and precision), in addition to the datasets for each system. Additionally, a comparison between the practicality and reliability of each of the four DDD categories is presented. Additionally, the paper covers the recent challenges in the DDD area. Furthermore, we discuss the DDD’s future trends and research directions that utilize smartphones, edge computing, and the Internet of Things (IoT).

This paper is organized as follows: Section 2 discusses drowsiness stages and signs. Section 3 provides a detailed investigation of driver drowsiness measures. These measures are categorized as image-, biological-, vehicle-, and hybrid-based. Section 4 covers a list of the challenges facing DDD. Section 5 compares the practicality and reliability of the four DDD system types and discusses the measures and methods, as presented in Section 3. Section 6 discusses some of the future trends in drowsiness detection systems. Finally, Section 7 concludes the paper.

## 2. Drowsiness Signs and Stages

In the literature concerning the design of drowsiness detection systems, different terms of reference are used. Although “drowsiness” is the commonly mentioned term, “fatigue” is also used. Despite their difference, fatigue and drowsiness are interchangeably utilized [14]. Fatigue refers to “the reluctance to continue a task as a result of physical or mental exertion or a prolonged period of performing the same task” [15]. However, sleepiness or drowsiness is defined as the urge to fall asleep. Basically, drowsiness is the result of a captivating biological need to sleep [15]. Drowsiness can happen due to many reasons, such as medication, working for long hours, sleep disorders, poor quality (or not having enough) sleep, and being awake for long periods [15]. Thus, their relationship is evident, as fatigue directly contributes to drowsiness. Although they are different concepts, some researchers considered drowsiness and fatigue alike, due to their similar consequences, such as [15,16,17,18]. In our work, we refer to these systems as drowsiness detection systems.

A driver does not become drowsy suddenly, without showing some signs. Examples of such signs include [6,13]:

- Difficulty keeping eyes open;
- Yawning;
- Frequent blinking;
- Difficulty concentrating;
- Swerving out of the lane and delayed reaction to traffic;
- Nodding;
- Unjustifiable variations in speed.

These signs gradually become more apparent as drowsiness deepens and, as such, can serve as indicators for the level of driver drowsiness. 

To systematically evaluate stages of drowsiness and facilitate the development of automatic early drowsiness detection systems, a precise measurement scale for drowsiness levels is necessary. Many methods have been proposed in that direction. One of the widely used scales in the literature is the Karolinska sleepiness scale (KSS) [19,20,21]. Shahid et al. define KSS as “a scale that measures the subjective levels of sleepiness at a particular time during the day” [22] (p. 209). KSS is a nine-point scale that measures drowsiness through verbal descriptions of drivers [19]. The nine KSS scores are summarized in Table 1. 

Wierwille and Ellsworth proposed another drowsiness evaluation scale [23]. They define drowsiness stages on a five-level scale, as shown in Table 2. According to Saito et al., at level one, rapid eye movement and a stable eye blinking period can be observed [24]. At level two, slow eye movement occurs. The driver may touch his face at level three, as well as yawn and slowly blink. As for level four, the driver’s unnecessary movements are observed; he frequently yawns, blinks more, and breathes deeply. Finally, the eyes are almost closed at the fifth level, and the head nods. 

This scale is also widely used because these levels are determined based on analyzing the driver’s facial expressions. When comparing this scale results with the subjective reports of the drivers, they show a high correlation, which indicates that this evaluation scale could be an alternative to the KSS scale [24,25].

## 3. Drowsiness Detection Measures

**Figure 1:**
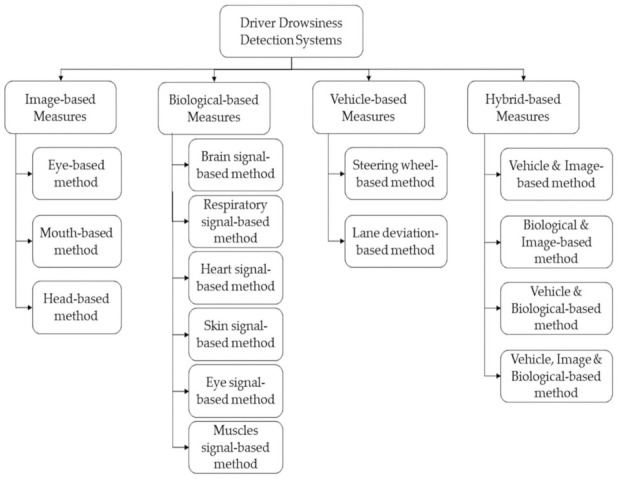
Driver drowsiness detection measures.

In order to detect the different stages of drowsiness, researchers have studied driver responses and vehicle driving patterns. In this section, we provide a review of the four widely used measures for DDD. The diagram in Figure 1 illustrates all the currently used measures for classifying driver drowsiness levels. Two of these measures are observed in the drivers themselves: image- and biological-based. The third measure is extracted from the car itself and referred to as the vehicle-based measure. The fourth measure considered is the hybrid measure, which combines at least two of the previously mentioned ones.

**Figure 2:**
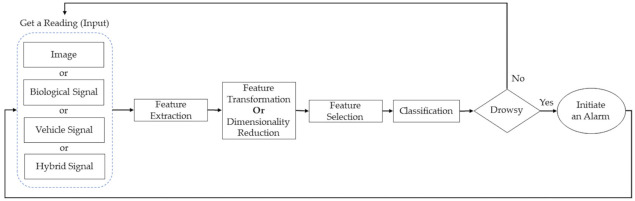
Driver drowsiness detection systems data flow.

Figure 2 illustrates a DDD system’s general block diagram and data flow that can employ any of the four measures mentioned above. Initially, data are captured using a suitable sensing device; then, the target features are extracted from the captured signals. This step is essential because it simplifies the system input by discarding irrelevant information and extracting useful ones. Next, some systems may employ feature transformation or dimensionality reduction, in order to project the data in another domain, where it is easier to analyze or reduce the computational load. The fourth step selects the features that best correlate to drowsiness, using different feature selection algorithms, such as backward selection or wrapper feature selection methods. After that, machine learning (ML) or deep learning is utilized to generate a model in the training phase that is used to classify the driver’s status. The trained model is used in the testing phase to detect the driver’s drowsiness level and, if required, take action, such as activating an alarm or alerting the driver to take a break. 

Various metrics have been used to evaluate the ability of the system to detect drowsy subjects. These include accuracy, precision, and sensitivity. The equations for three metrics are listed below (1)–(3) [26,27].

(1) $$\mathrm{Accuracy}=\frac{\mathrm{Number}\;\mathrm{of}\;\mathrm{correct}\;\mathrm{predectins}}{\mathrm{Total}\;\mathrm{number}\;\mathrm{of}\;\mathrm{redections}}=\frac{\mathrm{TP}+\mathrm{TN}}{\mathrm{TP}+\mathrm{TN}+\mathrm{FP}+\mathrm{FN}}$$

(2) $$\mathrm{Precision}=\frac{\mathrm{TP}}{\mathrm{TP}+\mathrm{FP}}$$

(3) $$\mathrm{Sensitivity}=\frac{\mathrm{TP}}{\mathrm{TP}+\mathrm{FN}}$$

TP (true positive) is the number of drowsy drivers that the system has correctly identified as drowsy, and TN (true negative) is the number of alert drivers that the system has correctly identified as alert. On the other hand, FP (false positive) is the number of alert drivers that the system has wrongly identified as drowsy, and FN (false negative) is the number of drowsy drivers that the system has wrongly identified as alert. 

Accuracy, which is the most commonly used metric, is a good indicator of how well the system can identify both TP and TN. However, it is more suitable when the data are balanced, i.e., when the number of drowsy drivers in an experiment equals the number of alert drivers in the same experiment. Otherwise, accuracy will be biased towards the class with more samples or data points. In many cases, it is easier to obtain awake driver data than it is to get drowsy driver data. In a real-life scenario, more awake drivers are on the road than drowsy ones. Therefore, to avoid bias, precision and sensitivity, also known as recall, are better alternatives for unbalanced datasets. 

Precision shows a proportion of the correctly identified drowsy drivers to those labeled as drowsy, while they are, in reality, alert. In contrast, sensitivity shows a proportion of the correctly identified drowsy drivers to those labeled as alert, while, in reality, they are drowsy. Low precision indicates that the system may identify alert drivers as drowsy and take actions to alert them. In contrast, low sensitivity means that the system may not be able to identify drowsy drivers, which could lead to serious accidents. It is, therefore, essential to have high sensitivity in DDD systems.

Other factors that are considered in the comparison of the four system types are cost, invasiveness, intrusiveness, and ease of use. Ease of use refers to the complexity of setting up the system at the beginning of each trip. All of these factors are covered here, under the umbrella of practicality. Generally, a trade-off between the system’s performance and cost must be weighed.

### 3.1. Image-Based Measures

Some drowsiness signs are visible and can be recorded by cameras or visual sensors. They include the driver’s facial expressions and movements, especially the head movements. The literature refers to these signs as visual [8] or image-based measures [7]. Our work refers to them as image-based measures to highlight that these measures usually lead to features extracted from images or videos. Additionally, it is important to note here that image-based measures are a subcategory of the physical [10] or behavioral measures [9]. Physical and behavioral measures refer to the body movements captured either from videos or using motion sensors, such as a gyroscope and accelerometer [28,29]. 

Image-based DDD systems can be broadly categorized into three techniques, based on whether movements of the mouth, head, or eyes are observed. Table 3 lists some of the image-based measures.

One widely used dataset among the Image-based DDD systems is the National Tsing Hua University Drowsy Driver Detection (NTHUDDD) public dataset by the Computer Vision Lab of National Tsing Hua University [35]. This dataset gained popularity due to the various scenarios and drowsiness features it covers. The dataset includes training, evaluation, and testing datasets and contains recorded videos for 36 subjects from different ethnicities. Additionally, it considers the cases when the driver is wearing sunglasses/glasses, day and night illumination conditions, and a variety of simulation scenarios, including:

- Normal driving;
- Yawning;
- Slow blink rate;
- Falling asleep;
- Burst out laughing.

The training dataset includes videos for 18 subjects in five different scenarios, including subjects with (1) bare face, (2) glasses, (3) bare face at night, (4) glasses at night, and (5) sunglasses. The videos include the two most important scenarios. Firstly, a combination of drowsiness symptoms, such as slow blink rate, yawning, and nodding. Secondly, a variety of non-drowsiness actions, such as talking, looking at both sides, and laughing. On the other hand, the testing and evaluation datasets contain videos from the remaining 18 subjects. These videos include drowsy and non-drowsy features, mixed under multiple scenarios.

Below, we discuss some image-based detection systems that have been introduced over the past decade. Table 4 provides a summary of those systems.

1. Fatigue detection, based on awning in thermal images

In their paper, Knapik and Cyganek presented a novel approach for driver fatigue detection, based on yawning detection, using long-range infrared thermal imaging [16]. A special dataset was created for this research [36]. The system works as follows. First, images are acquired from a thermal video. Then, three cascaded detection modules are applied for the face area, eye corners, and yawn. Since the mouth area is sometimes hard to detect in thermal images, due to the temperature difference in that area, information about other face regions’ relative temperatures is used to detect the yawn reflex. Thus, the authors used the eye corners as an indicator for yawning. Cold and hot thermal voxel sum methods were used to detect yawning [37]. Finally, based on the proposed algorithm’s results and assumed constraints, an alarm is initiated when fatigue is detected. The system showed accuracies of 71% for cold voxels detection and 87% for hot voxels detection.

2. Drowsiness detection using respiration in thermal imaging

Kiashari et al. [38] introduced a non-intrusive system that detects drowsiness using facial thermal imaging to analyze the driver’s respiration signal. Thirty subjects participated in their study, which was conducted in a car simulator. A thermal camera was used to capture the driver’s thermal images. From the obtained thermal signals, the standard deviation and mean of both the respiration rate and inspiration-to-expiration time ratio were calculated and used as input features, in order to train two machine learning classifiers, namely, support vector machine (SVM) and k-nearest neighbor (KNN). Both classifiers were able to detect drowsiness. However, SVM outperformed the KNN, with 90% accuracy, 85% specificity, 92% sensitivity, and 91% precision.

3. Drowsiness detection using eye features

  - Eyelid closure analysis

Khan et al. [39] proposed a real-time DDD system based on eyelid closure. The system was implemented on hardware that used surveillance videos to detect whether the drivers’ eyes were open or closed. The system started by detecting the face of the driver. Then, using an extended Sobel operator, the eyes were localized and filtered to detect the eyelids’ curvature. After that, the curvature’s concavity was measured. Based on the measured concavity value, the eyelid was classified as open (concave up) or closed (concave down). If the eyes were deemed closed for a certain period, a sound alarm is initiated. The system used three datasets. The authors generated two of them, and the third was acquired from [40]. The first dataset, which contained simple images, with a homogenous background, showed an accuracy of 95%. The second set, which included a complex benchmark image dataset, achieved an accuracy of 70%; the third one, which used two real-time surveillance videos, showed an accuracy that exceeded 95%.

  - Optical correlator based DDD algorithm

Ouabida et al. [41] proposed a fast method for DDD that depends on an optical correlator to detect the eye and then estimates its state using optical correlation with a deformed filter. This method was the first to use a numerical simulation of the optical Vander Lugt correlator [42,43] to detect the eye center automatically. The proposed DDD method precisely estimates the eye’s location and state (open or closed), using a specific filter in the Fourier plane of the optical Vander Lugt correlator. In this method, the eyes are initially detected in non-zoomed facial images. Using the simulated optical correlator, the eye state is estimated under different lighting, head orientations, and with or without eyeglasses. The researchers evaluated the proposed method on five international databases: FEI [44], ICPR [45], BioID [46], GI4E [47], and the second Strategic Highway Research Program results (SHRP2) [48]. Additionally, a group of correlation filters was proposed and designed to recognize eyes’ states in noisy and cluttering environments. The proposed optical correlation, with a deformed eye filter, showed the best performance.

  - Real-time DDD using eye aspect ratio

In this work, Maior et al. [49] developed a drowsiness detection method based on eye patterns monitored by video streams using a simple web camera. The method tracks the blinking duration using the EAR metric. The proportion between the eye’s height and width is calculated to evaluate the EAR value. A high EAR value indicates that the eye is open, while a low value indicates that it is closed. The proposed method consists of three main parts: eye detection, EAR calculation and blink classification, and real-time drowsiness detection. An experiment was conducted to generate a training database. After obtaining the images from the web camera, the EAR values were calculated and stored for each frame. Then, a specific number of consecutive values were used as input for the machine learning algorithms. Drowsiness is detected if the blink duration is longer, compared to a standard blink. Three classification methods were employed: multilayer perceptron, random forest (RF), and SVM. Overall, SVM showed the best performance, with an average test accuracy of 94.9%.

  - DDD using face and eye features

In [30], Bamidele et al. presented a nonintrusive DDD system, based on face and eye state tracking. The research utilized the NTHUDDD Computer Vision Lab’s video dataset [35]. The proposed system starts by acquiring and pre-processing the required data. Then, it extracts the targeted features, including the PERCLOS, maximum closure duration of the eyes, and blink frequency. The extracted features are then fed to various classifiers to decide whether they belong to a drowsy or awake person. These classifiers include KNN, SVM, logistic regression, and artificial neural networks (ANN). The final results revealed that the best models were the KNN and ANN, with accuracies of 72.25% and 71.61%, respectively.

  - Detection of driver drowsiness with CNN

Hashemi et al. proposed a real-time DDD system based on the area of eye closure and use of the convolutional neural network (CNN) [50]. Three networks were introduced for eye closure classification: fully designed neural network (FD-NN), transfer learning in VGG16 (TL-VGG16), and transfer learning in VGG19 (TL-VGG19), with extra designed layers. The authors used the ZJU gallery dataset, in addition to 4157 new images. The experiment resulted in the following network accuracies: 98.15%, 95.45%, and 95%, respectively.

  - Eye signal analysis

Zandi et al. [51] proposed a non-intrusive drowsiness detection ML system based on eye-tracking data. The experiments were conducted in a simulated driving environment, with 53 participants. The authors collected data for eye-tracking signals and multichannel electroencephalography signals. The electroencephalography signal was only used as a reliable baseline for comparison and to label the eye-tracking signals epochs as drowsy or alert. The proposed ML system extracted 34 eye-tracking signals’ features, obtained from overlapping eye signals’ epochs with different lengths. The system performance, subject to various combinations of different features and epoch lengths, was also studied. Two binary classifiers were used: the RF classifier with 200 trees and non-linear SVM with a Gaussian kernel classifier. The experiment results revealed that the RF classifiers resulted in an accuracy range of 88.37% to 91.18% across all epochs, as well as a sensitivity–specificity of 88.1% to 88.8% for a 10-s epoch. In contrast, the non-linear SVM classifier showed an accuracy range of 77.12% to 82.62%. Additionally, it resulted in a sensitivity–specificity of 79.1% to 80.8% for a 10-s epoch. Using eye-tracking data and a proper classification framework, such results confirmed that drowsiness could be reliably detected with high accuracy, specificity, and sensitivity.

4. Drowsiness detection using multiple features

  - Eye and mouth analysis

Celecia et al. [52] proposed a low-cost, portable, robust, and accurate DDD device that used an infrared illuminator and camera to record images. The device’s processing model, which was performed over a Raspberry Pi 3 Model B, combines features obtained from the eyes and mouths of the subjects under consideration. The features include PERCLOS [31], eye closing duration, and average mouth opening time. The 300-W dataset [53] was used in the training process. The authors determined the state of each feature through a cascade of regression tree algorithms. A Mamdani fuzzy inference system then estimated the driver state by combing the three features’ states as an input. The device generates a final output that represents the drowsiness level by giving a label of either “Low-Normal”, “Medium-Drowsy”, or “High-Severe state.” According to Celecia et al., using various drowsiness measures overcomes the issues of partly losing some of them in the image. Thus, the study resulted in a DDD device, robust to different ambient lighting conditions, with 95.5% accuracy.

  - Eye state analysis and yawning

Alioua et al. [54] proposed a non-intrusive and robust system that detects drowsiness in real-time to reduce traffic accidents. The system detects drowsiness based on a closed-eyes and open-mouth detection algorithm. In this work, a group of images was collected using a webcam. According to the authors, the system starts with an SVM face detector to extract the face region from the video frames. Then, the eye and mouth regions localization within the face is performed. Finally, the circular Hough transform is applied to the extracted eye to find the iris, a colored muscular curtain close to the front of the eye, as an indication of eye openness. Additionally, it is used over the mouth region to determine the degree of mouth openness. Based on the fusion of the state of the eye and the mouth, the system decides whether the driver is drowsy or not. The results showed that this system is robust, with 94% accuracy and 86% kappa statistic value.

  - Eye Closeness

In order to detect the levels of drowsiness, Khunpisuth et al. [55] conducted a study with ten volunteers. During the study, the frequency of eyes blinking and head tilting was monitored and related to the drivers’ drowsiness state. The authors built an embedded device for drowsiness detection that used a Raspberry Pi Camera and Raspberry Pi 3 Model B to collect image data, detect the drowsiness level, and alert the driver. Initially, the proposed device applied the Haar cascade classifier to detect an upright face, head level, and eye blinking. Moreover, if the head position is not upright, geometric rotation is used to calculate the angle and rotate the image to an upright position, in order to detect accurately. Secondly, template matching is used to detect whether the eyes are open or closed. Thirdly, the drowsiness level is calculated via the frequency of head tilting and eye blinking. The system uses a scale of 0–100 to describe the severity of the drowsiness. If the drowsiness level reaches 100, the system triggers a loud, audible warning to alert the driver. Finally, the accuracy system gave an accuracy of 99.59%. However, this system had some limitations, as it is affected by the subject’s skin tone and background light.

  - Facial features

Deng and Wu [56] proposed DriCare, a real-time DDD system. This system detects the drowsiness status using images from video streams. The authors introduced an enhanced in-video face-tracking algorithm, called multiple CNNs-kernelized correlation filters. Further, they used 68 key points in the driver’s face to locate key regions, including the eyes and mouth. The authors then calculated the number of closed-eye frames to the total number of frames, continuous-time of eye closure, blinking frequency, and number of yawns in a minute to detect the driver’s drowsiness. Finally, the DriCare system alerts the driver, using some warning, if found drowsy. The system was tested on CelebA [57], YawDD [58] datasets, and other videos obtained by the authors. Overall, the system showed an accuracy of around 92%.

  - Deep CNN models-based ensemble approach

Dua et al. [59] utilized the NTHUDDD public dataset [35] to propose an architecture that detects driver drowsiness. This architecture comprises of four deep learning models: AlexNet, VGG-FaceNet, FlowImageNet, and ResNet. These models are used to extract four different types of features: facial expression, head gestures, hand gestures, and behavioral features, such as head, eyes, or mouth movements. While the AlexNet model accounts for different environmental and background conditions, the VGG-FaceNet model detects and extracts facial traits. In contrast, FlowImageNet is used to extract head gestures and behavioral features, while ResNet is used for hand gestures. Using RGB videos of the drivers as an input, the four models generate four outputs that are fed to an ensemble algorithm, called simple averaging [60], followed by a SoftMax classifier [61]. Dua et al. proposed system resulted in an overall accuracy of 85%.

  - Fatigue detection using convolutional two-stream network

Liu et al. [17] presented a fatigue detection algorithm that feeds multi-facial features, such as eye closure duration, head nodding, and yawning, to a convolutional two-stream network, referred to as a gamma fatigue detection network. Initially, the algorithm locates the eyes and mouth of the driver using multi-task cascaded CNNs. The static features are then extracted from a partial facial image. After that, the dynamic features are extracted from a partial facial optical flow. Once obtained, both static and dynamic features are combined using a two-stream neural network to classify the image data. In addition, the paper showed that applying gamma correction [62] to enhance image contrast increased the accuracy by 2% for night shoots. The algorithm was verified using the NTHUDDD public dataset [35], with an accuracy of 97.06%.

  - Condition-adaptive representation learning

Yu et al. [63] presented a condition-adaptive representation learning framework for DDD, based on a 3D-deep CNN using the NTHUDDD public dataset. The framework contained four models: spatio-temporal representation learning, scene condition understanding, feature fusion model, and the drowsiness detection model. 

First, spatio-temporal representation learning was used to simultaneously extract features that describe movements and appearances in the video. Then, scene condition understanding was used to represent different driving conditions and classify the drivers. Such conditions include facial changes in the eye, mouth, and head, in addition to others. Then, the feature fusion model generates an adaptive representation for driving conditions by fusing two features. Finally, the drowsiness detection model recognizes the drivers’ alertness status, using the condition-adaptive representation from the previous model. The framework’s accuracy was 76.2%.

  - Face descriptors

Moujahid et al. introduced a face monitoring DDD system that can capture the most discriminant drowsiness features [33]. It is based on a hand-crafted, compact face texture descriptor. After extracting the raw features, the compactness is achieved by employing pyramid multi-level face representation and feature selection. This work used the NTHUDDD [35] public dataset. The authors have focused on extracting the tiredness features from the eyes, head, and mouth, such as blinking rate, head nodding, and yawning frequency. This process led to three descriptors, namely covariance descriptor [64], a histogram of oriented gradients features [65], and classical texture local binary pattern features [66]. The framework consists of five phases: first, face detection and alignment; second, pyramid multi-level face representation; third, pyramid multi-level feature extraction; fourth, dimensionality reduction principal component analysis and subset feature selection, using the Fisher score [67]; finally, non-linear SVM-based classification. After testing the data with several DDD methods, the experimental results showed that the proposed method achieved an accuracy of 79.84%. Furthermore, these results proved that this method is similar or superior to other approaches that rely on deep CNN.

  - Facial motion information entropy

In [18], You et al. proposed a real-time algorithm for driver fatigue detection using facial motion information entropy. The algorithm contains four modules. First, a face positioning module, where the authors presented an improved YOLOv3-tiny CNN to capture the facial regions, under various complex conditions, within the captured video frames. The second module is dedicated to feature vector extraction. In this module, a face feature triangle geometry area was constructed using the Dlib toolkit, face’s landmarks, and facial regions’ coordinates. The third module involves extracting face feature vectors that contain information about each face feature triangle area, as well as the centroid extracted for each frame. This vector is used as an indicator to determine the driver’s state. In the fourth module, the fatigue judgment module, a sliding window is designed to acquire the facial motion information entropy. This information is then compared to a judgment threshold, specified by the SVM classifier, to evaluate the driver’s fatigue state. The authors verified their proposed algorithm using an open-source dataset (YawDD [58]). You et al. reported accuracy of 94.32%.

  - Monitoring drowsiness on a mobile platform

Wijnands et al. [68] described a new DDD method, based on activity prediction, through depth-wise separable 3D CNN using real-time video. Similar to others, their method used the academic NTHUDDD dataset [35]. An advantage of this method is that it implicitly decides on the essential features, rather than pre-specifying a set of features beforehand. Some features include eyelid closure, mouth position, frowning, outer brow raises, nose wrinkles, and chin raises. Thus, if a sufficient amount of data labels are provided, it will capture these features. The experiments were conducted under different lighting and face wear conditions, including driving at night and daytime. Additionally, subjects drove while wearing glasses, sunglasses, and without any. The results presented different accuracies, based on the different scenarios and selected features, but the method showed a final accuracy of 73.9%.

  - DDD with hybrid CNN and LSTM

Guo and Markoni [69] proposed a new method that applies real-time DDD, based on a combination of CNN and long short-term memory (LSTM). The proposed method consists of two parts: spatial and temporal. In the spatial part, the method extracts facial features, such as eyes and mouth, in one frame. CNN was used for face detection, face landmark detection, and eyes and mouth classification. As for the temporal part, an LSTM analyzer used the concatenated spatial features that indicate drowsiness or alertness for analysis and final classification. 

Overall, the DDD method follows three steps. First, face detection using multi-task cascaded CNN and landmark extraction, along with spatial feature extraction, which is done by utilizing CNN. Then, temporal features are formed by concatenating spatial features through frame vector concatenation using sliding windows. Finally, the concatenated features are fed to an LSTM, where a decision of drowsiness (or not) is made. This method employed the NTHUDDD public dataset from the ACCV 2016 competition [35]. Various accuracies for the different applied scenarios and experiments were presented. However, the proposed method gave a final accuracy of 84.85%.

  - Fatigue detecrion using new CNN method

Ed-Doughmi et al.’s research [70] presented an approach to analyze and predict fatigue based on a recursive neural network (RNN), using a sequence of frames from videos. The authors implemented a repetitive neural network architecture, based on an RNN model, called multi-layer, model-based 3D convolutional networks [71], to detect fatigue. They detected fatigue by extracting the subjects’ drowsy behaviors, such as yawning, eye closure, and head nodding, from the NTHUDDD dataset videos. An accuracy of 97.3% was obtained [35].

  - Fatigue detection using eye and mouth CNN

Zhao et al. proposed a fully automated driver fatigue detection algorithm [72]. This study uses the driving images dataset provided by Biteda, an information technology company. This algorithm applies face detection and feature points location, using a multitask cascaded CNN architecture, where the region of interest (ROI) can be extracted using the feature points. Moreover, a new CNN algorithm, called eye and mouth CNN (EM-CNN), was proposed. The EM-CNN algorithm detects the mouth and eye state from the ROI. Both the PERCLOS and mouth opening degree were used as parameters for detection. The final results showed an accuracy of 93.62% and sensitivity of 93.64%.

Table 4 reveals that image-based systems have reported accuracies between 72.25% and 99.59%, with [55] showing the highest accuracy. Most of them rely on eye state features. Generally, such systems are non-intrusive, non-invasive, and cost-effective, as they require only a camera to collect the needed data. However, the system’s performance is severely affected in cases where it is difficult to track facial data due to obstacles. Further details are discussed later in the challenges section.

**Table 4:** Image-based drowsiness detection systems.

Ref.	Image-Based Parameters	Extracted Features	Classification Method	Description	Quality Metric	Dataset
[16]	Mouth	Yawning	Cold and hot voxels [37]	A fatigue detection method based on yawning detection using thermal imaging. The cold and hot voxels were used to detect yawning.	Accuracy: Cold voxels: 71%, Hot voxels: 87%	Prepared their own dataset [36]
[38]	Respiration (using thermal camera)	Standard deviation and the mean of respiration rate, as well as the inspiration-to-expiration time ratio	SVM and KNN	Used facial thermal imaging to study the driver’s respiration and relate it to drowsiness.	Accuracy: SVM: 90%, KNN: 83% Sensitivity: SVM: 92%, KNN: 82% Precision: SVM: 91%, KNN: 90%	New thermal image dataset was prepared
[39]	Eye	Eyelids’ curvature	Classification based on the period of eye closure	Based on the eyelid’s curvature’s concavity, the system determined if the eye is opened or closed. Then, it detected drowsiness based on the eye closure period.	Accuracy: Dataset 1: 95%, Dataset 2: 70%, Dataset 3: >95%	Dataset1: Prepared their own image dataset Dataset2: Benchmark dataset [40] Dataset3: Prepared their own video dataset
[41]	Eye	Eye state (open/closed)	Proposed optical correlation with deformed filter	Used optical Vander Lugt correlator to precisely estimate the eye’s location in the Fourier plane of the Vander Lugt correlator.	Different accuracies for different datasets	FEI [44], ICPR [45], BioID [46], GI4E [47], and SHRP2 [48]
[49]	Eye	The eyes’ EAR value	Multilayer perceptron, RF, and SVM	Tracked eye blinking duration in video streams, as an indicator of drowsiness using the EAR. Overall, the SVM showed the best performance.	Accuracy: SVM: 94.9%	Prepared their own dataset
[30]	Face and eye	PERCLOS, blink frequency, and maximum closure duration of the eyes.	KNN, SVM, logistic regression, and ANN	A nonintrusive system based on face and eye state tracking. The final results revealed that the best models were the KNN and ANN.	Accuracy: KNN: 72.25% ANN: 71.61% Sensitivity: KNN: 83.33% ANN: 85.56%	NTHUDDD public dataset [35]
[50]	Eye	Eye closure	FD-NN, TL-VGG16, and TL-VGG19	Applied real-time system based on the area of eye closure using CNN. For eye closure classification, three networks were introduced: FD-NN, TL-VGG16, and TL-VGG19.	Accuracy: FD-NN: 98.15%, TL-VGG16: 95.45%, TL-VGG19: 95%	ZJU gallery and prepared their own dataset
[51]	Eye	34 eye–eye tracking features	RF and non-linear SVM	Used 34 eye-tracking signals’ features to detect drowsiness. These features were extracted from overlapping eye signals’ epochs of different lengths. The labels were extracted from EEG signals.	Accuracy: RF: 88.37% to 91.18% SVM: 77.1% to 82.62% Sensitivity for 10s epoch: RF: 88.1% SVM: 79.1%	Prepared their own dataset
[52]	Eye and Mouth	PERCLOS, eye closing duration, and average mouth opening time	Mamdani fuzzy inference system	The state of the extracted parameters is determined through a cascade of regression tree algorithms. A Mamdani fuzzy inference system then estimates the driver state.	Accuracy: 95.5% Precision: 93.3%	300-W dataset [53]
[54]	Eye and Mouth	Eye closure and mouth openness for a duration of time	Circular Hough transform	The circular Hough transform method is applied to check whether the mouth is open or iris is detected. Based on these two measures, the driver’s state is determined.	Accuracy: 94%	Prepared their own dataset
[55]	Eye and Head	Frequency of eyes blinking and frequency of head tilting	Templet matching to detect the eyes and calculating the frequency of head tilting and eye blinking to detect the drowsiness level	By calculating the frequency of head tilting and eye blinking, the drowsiness level is determined, on a scale of 0-100. If drowsiness reached 100, a loud audible warning would be triggered.	Accuracy: 99.59% Precision: 97.86%	Prepared their own dataset
[56]	Mouth and Eye	Proportion of the number of closed-eye frames to the total number of frames in 1min, continuous-time of eye closure, blinking frequency, and number of yawns in 1-min	For face tracking: multiple CNNs-kernelized correlation filters method For drowsiness detection: newly proposed algorithm	The multiple CNNs-kernelized correlation filters method is used for face tracking and to extract the image-based parameters. If found drowsy, the driver is alerted.	Accuracy: 92%	CelebA dataset [57], YawDD dataset [58], and new video data were prepared
[59]	Facial, hand, Behavioral (head, eyes, or mouth movements)	Facial expression, behavioral features, head gestures, and hand gestures	SoftMax classifier	This system introduced an architecture that uses four deep learning models to extract four different types of features.	Accuracy: 85% Sensitivity: 82% Precision: 86.3%	NTHUDDD public dataset [35]
[17]	Eye, head, and mouth	Eye closure duration, head nodding, and yawning	A two-stream CNN	Used multi-task cascaded CNNs to find the positions of the mouth and eyes. Then, it extracted the static and dynamic features from a partial facial image and partial facial optical flow, respectively. Lastly, it combined the features to classify the image data.	Accuracy: 97.06% Sensitivity: 96.74% Precision: 97.03%	NTHUDDD public dataset [35]
[63]	Eye, mouth, head, and scene conditions	Facial changes in eye, mouth, and head, illumination condition of driving, and wearing glasses	3D-deep CNN	The framework contained four models to recognize the drivers’ alertness status, using the condition-adaptive representation.	Accuracy: 76.2%	NTHUDDD public dataset [35]
[33]	Eye, head, and mouth	Blinking rate, head-nodding, and yawning frequency	Fisher score for feature selection and non-linear SVM for classification	The system is based on a hand-crafted compact face texture descriptor that can capture the most discriminant drowsy features.	Accuracy: 79.84%	NTHUDDD public dataset [35]
[18]	Facial features	Face feature vectors	SVM	Used facial motion information entropy, extracted from real-time videos. The algorithm contained four modules.	Accuracy: 94.32%	YawDD dataset [58]
[68]	Facial features, head movements	Implicitly decides the important features like eye closure, mouth position, chin or brow raises, frowning, and nose wrinkles	3D CNN	DDD was performed, based on activity prediction, through a depth-wise separable 3D CNN, using real-time face video. An advantage of this method was that it implicitly decided the important features, rather than pre-specifying a set of features beforehand.	Accuracy: 73.9%	NTHUDDD public dataset [35]
[69]	Eye and mouth	Temporal facial feature vectors formed from spatial features	LSTM	A method that applied real-time DDD, based on a combination of CNN and LSTM. It consisted of two parts: spatial and temporal.	Accuracy: 84.85%	NTHUDDD public dataset [35]
[70]	Eye, head, and mouth	Yawning, eye closure, and head nodding	Multi-layer model-based 3D convolutional networks	Used a repetitive neural network architecture, based on an RNN model, called multi-layer model-based 3D convolutional networks, to detect fatigue.	Accuracy: 97.3% Sensitivity: 92% Precision: 72%	NTHUDDD public dataset [35]
[72]	Eye and mouth	PERCLOS and mouth opening degree	Eye and mouth CNN	Applied face detection and feature points location, using multi-task cascaded CNNs architecture and EM-CNN to detect the mouth and eye state from the ROI.	Accuracy: 93.62% Sensitivity: 93.64%	Driving images dataset from Biteda company

### 3.2. Biological-Based Measures

Many biological signals have been used to detect the driver’s drowsiness, such as brain activity, heart rate, breathing rate, pulse rate, and body temperature signals [10]. These biological signals, also known as physiological measures [9], are proven to be more accurate and reliable for detecting drowsiness. This accuracy is due to their ability to capture early biological changes that may appear, in the case of drowsiness, thus alerting the driver before any physical drowsiness signs appear. The most commonly used biological measures in literature are listed in Table 5.

This section will cover some of the systems that detect drowsiness using the driver’s biological changes. A summary of these systems is shown in Table 6.

1. Drowsiness detection using EEG signals

The EEG signals reveal brain activities. They provide valuable information about brain physiology. Such an approach has gained extra attention in the past years because EEG signals can show the changes in the brain activity of a drowsy driver, allowing for early detection of drowsiness.

  - Smartwatch-based wearable EEG system

Li et al. [79] proposed a driver drowsiness detection system based on EEG signals. The proposed system employs an SVM-based posterior probabilistic model for drowsiness detection, in order to classify the drowsiness states into three categories (alert, drowsy, and early warning). This method is slightly different from other EEG-based detection systems, which generate discrete drowsiness labels, identifying the driver’s state as drowsy or alert. Thus, instead of using discrete labels to identify the driver’s drowsiness level, the SVM-based posterior probabilistic model transforms the drowsiness level to a value between 0 and 1, providing a continuous measure for drowsiness. This work’s fully wearable EEG system included a commercial smartwatch and a Bluetooth-enabled EEG, enabling real-time data evaluation. This system showed different accuracies for each detected state. It obtained a 91.92% accuracy for the drowsy case, 91.25% for the alert case, and 83.78% for the early warning case.

  - EEG signal analysis using EMD and trained neural network

Kaur and Singh [80] presented a method to detect driver drowsiness, based on EEG signal analysis, using empirical mode decomposition (EMD) and trained ANN. Kaur and Singh placed silver surface electrodes on the subject’s scalp to extract the EEG signals. In addition, they have used a video camera to provide a drowsiness label, alongside the EEG features. Thus, they produced their own dataset. Then, drowsiness positions in the EEG signals were labeled as drowsy or awake using a utility designed in MATLAB. Afterward, using the EMD method, the intrinsic mode functions (IMFs) were obtained from the labeled EEG data. Finally, the IMFs were used as an input to train the ANN. A total of 70% of samples were used for training, 15% for testing, and 15% for validation. The final classification results showed an accuracy of 88.22%.

  - EEG features with LTSM

Budak et al. [81] proposed an EEG-based drowsiness detection method that consists of three essential building blocks. The instantaneous frequency and spectral entropy features are extracted from the EEG spectrogram images in the first block. The raw EEG signals are analyzed, as well, to calculate the energy distribution and zero-crossing distribution features. In the second block, using pre-trained AlexNet and VGG16 models, in-depth features are directly extracted from the EEG spectrogram images. As for the third block, the EEG signals are decomposed into related sub-bands, through a tunable Q-factor wavelet transform. The authors then calculate the obtained sub-bands spectrogram images and statistical features, such as the sub-bands instantaneous frequencies’ mean and standard deviation. After processing the three blocks, the extracted feature groups are fed to an LSTM network classifier. The method was trained and evaluated on MIT/BIH polysomnographic EEG dataset [82]. Specifically, a subset was collected from 16 subjects, with ages and weights of around 43 years and 119 kg, respectively. Finally, the proposed method was evaluated using a 10-fold cross-validation test, obtaining a final average accuracy of 94.31%.

  - Adaptive Hermite decomposition and ELM

Taran and Bajaj [83] presented a DDD method, based on an adaptive Hermite decomposition for EEG signals. In general, Hermite functions help find applications for analyzing nonstationary and complex signals. In this decomposition, the Hermite functions were employed as basic functions, which were selected adaptively using evolutionary optimization algorithms for each EEG signal. The authors used the MIT/BIH polysomnographic database [82] in their research. The extracted features were taken from the statistical measures of Hermite coefficients, which were first quartile, median, range, and energy. These features were then tested and classified using the extreme learning machine (ELM) [84], KNN, decision tree, least-squares SVM, naive Bayes, and ANN classifiers. The ELM classifier obtained the highest accuracy, which was 92.28%.

  - Wired- and wireless-based EEG system

Choi et al. [85] presented a framework for detecting instantaneous drowsiness, with only 2-s EEG signal segments. Multi-taper power spectral density [86] was employed for feature extraction, and an extreme gradient boosting classifier was used for classification. This research defined a novel phenotype labeling method for detecting instantaneous drowsiness. Thus, the labeling was done by combining the psychomotor vigilance task’s advantages as a standard reference and EOG as a task-independent alertness measure. The framework was implemented on a wireless and wired EEG, in order to show the applicability of this mobile environment. The final results showed that the wired EEG gave an accuracy of 78.51%. At the same time, the wireless EEG gave an accuracy of 77.22%. This degradation in the performance is due to the instability of the wireless EEG dry sensors and small amount of EEG data used for training.

  - Wavelet packet transform employed on EEG

In [87], Phanikrishna and Chinara proposed a new drowsiness detection model that employs wavelet packet transform [88] to extract the time domain features from a single-channel EEG signal. The data used for this work was obtained from the Fpz-Cz channel dataset, a pre-recorded data available on the National Institute of Health [89,90]. Additionally, the simulated virtual driving driver (SVDD) dataset from [91] was utilized. Five sub-bands were extracted from the EEG signal: delta, theta, alpha, beta, and gamma. In the feature extraction stage, the Higuchi fractal dimension [92], mobility [93], and complexity characteristics of the EEG signal, in addition to the EEG sub-bands, extracted in the previous stage, were utilized to compute the values of nine features labeled from F1 to F9. Then, by applying the Mann–Whitney U test [94], followed by Wilkinson’s meta-analysis [95] method, the PComb values were computed for each feature. The features with the lower PComb values were selected for the last stage. Eleven classifiers were tested in this work. Out of the eleven classifiers, extra trees exhibited the best results, with an accuracy of 94.45% for the Fpz-Cz channel and 85.3% for the SVDD dataset.

  - Entropy-based detection using AVMD

In [96], Khare and Bajaj presented a drowsiness detection method that used adaptive variational mode decomposition (AVMD) to analyze and synthesize the EEG signals. This method utilized the MIT/BIH polysomnographic dataset [82]. Through the AVMD, the signal is decomposed into several modes. From the adaptively decomposed modes, the features were extracted. By applying statistical analysis, five entropy-based features were selected [97,98,99]: Tsallis entropy, Renyi entropy, permutation entropy, log energy entropy, and Shannon entropy. Then, ten classifiers were used to evaluate the classification accuracy. Among them, the ensemble boosted tree classifier achieved the highest results, with an accuracy of 97.19%.

2. Drowsiness detection using ECG, PPG, and HRV signals

ECG is a sensor that senses the heart’s electrical signals, indicating different heart conditions. In contrast, PPG is plethysmography used to detect the blood volume changes in the tissue’s microvascular bed. As for HRV, it refers to the variation in time between consecutive heartbeats.

  - Wearable ECG/PPG sensors

In 2019, Lee et al. [100] investigated driver’s drowsiness by tracking the distinguishable patterns of HRV signals. Such signals are obtained using wearable ECG or PPG sensors. According to the authors, wearable sensors tend to produce more noise in signals because they are vulnerable to slight movements. Thus, in order to classify the noisy HRV signals as drowsy or not, the authors explored three types of recurrence plots (RPs), obtained from the heartbeats’ R–R intervals (RRI). These RPs are the binary recurrence plot (Bin-RP), continuous recurrence plot (Cont-RP), and thresholded recurrence plot (ReLU-RP), which is acquired by using a modified rectified linear unit (ReLU) function to filter Cont-RP. Each recurrence plot is utilized as an input feature to a CNN. Then, the usefulness of each classification is examined. The study, conducted in a simulation environment, showed that DDD’s most reliable and distinct pattern was the ReLU-RP (using either the ECG sensor or the PPG sensor). ReLU-RP CNN could distinguish between awake and drowsy states better than the other alternatives. PPG signals gave 64% accuracy, 71% precision, 78% recall, and 71% F-score. On the other hand, ECG signals gave 70% accuracy, 71% precision, 85% recall, and 77% F-score. Overall, the ReLU-RP CNN showed an approximately 4 to 14% better accuracy for PPG and 6 to 17% for ECG in classification results, compared to the Bin-RP and Cont-RP results, respectively.

  - PPG biosignals and multimodal head support

Koh et al. [101] proposed a method for DDD by employing the high frequency (HF), low frequency (LF), and low to high frequency (LF/HF) values of the PPG signals measured from sensors mounted on fingers and earlobes. The experiments included 20 subjects aged, between the early twenties and late forties. The authors used a driving simulator equipped with two PPG sensors. A sensor was placed to touch the user’s earlobe, and the other was placed on the finger. The collected PPG signals were analyzed using Telescan and KITECH programs to design an algorithm to classify the driver’s drowsiness state. The classification relied on the changes in the extracted LF and HF values. The standard drowsy state criteria were specified by a decrease in LF and LF/HF values and increase in HF value. In contrast, other cases will indicate an awake driver. The results showed a significant difference in PPG signals in the two states.

  - DDD using wrist-worn wearable sensor

Kundinger et al. [102] proposed a non-intrusive retrofittable system that detects drowsiness, based solely on physiological data extracted from a wrist-worn wearable sensor. The study was conducted using a simulator, with over 30 subjects. First, the heart rate signals, including the ECG and PPG/ blood volume pulse, were collected and analyzed to get the HRV. Then, the HRV was used to obtain the autonomic nervous systems activity, which gave a more in-depth insight into the drowsiness status. Videos of the driver’s face were recorded to be used for labeling purposes. Multiple ML algorithms for binary classification were used, including random tree, RF, SVM, and decision stump, amongst others. KNN algorithm achieved the highest accuracy, around 92.13%.

  - HRV anomaly analysis

Fujiwara et al. [103] proposed an algorithm that uses HRV anomaly analysis to detect drowsiness, based on the fact that changes in alertness levels affect the autonomic nervous system and HRV. The HRV reflects this effect through the RRI fluctuation on the ECG trace. The R wave is the height peak on the ECG, and the RRI is the interval between two consecutive R waves. Using an anomaly detection method, referred to as the multivariate statistical process control method, Fujiwara et al. monitored changes in eight HRV features. These features include the mean of RRI (MeanNN), standard deviation of RRI (SDNN), root means square of the difference of adjacent RRI (RMSSD), total power (which is the variance of RRI) (TP), number of pairs of adjacent RRI spaced by 50 ms or more (NN50), LF, HF, and LF/HF. The proposed algorithm performance was evaluated experimentally in a simulator, with 34 participants. This algorithm was validated by comparing its results with EEG-based sleep scoring. The algorithm showed an accuracy of 92%.

3. Drowsiness detection using respiratory signals analysis

Respiratory signals can be used to provide information related to drowsiness. In fact, by tracking the diaphragm, abdomen, and rib cage changes during the respiratory process, the obtained signals can be linked to the driver’s drowsiness state.

Guede-Fernández et al. proposed a novel algorithm for DDD utilizing respiratory signal variations [104]. In their study, three respiratory inductive plethysmography band sensors were used to guarantee the best tracking quality of the respiratory signals. The study was conducted in a simulator cabin, with twenty volunteers, where 36 tests were done to collect the data. The proposed algorithm depends on analyzing the respiratory rate variability (RRV) to detect the driver’s alertness status changes. Furthermore, another method was used to ensure a quality level of the respiratory signals. Those two methods were combined to reduce the detection errors and formed the thoracic effort-derived drowsiness index algorithm. The system achieved a 90.3% sensitivity and 96.6% specificity.

4. Drowsiness detection using EMG signals

EMG is an electrodiagnostic medicine technique that is utilized to record and evaluate the electrical activities produced by the skeletal muscles [105]. EMG can be used for clinical or biomedical applications, modern human-computer interaction, and evolvable hardware chips [106]. The EMG signals can be analyzed and used to detect medical abnormalities and alertness levels, as well as to analyze the animal or human biomechanics movement.

  - Hypovigilance detection using higher-order spectra

Sahayadhas et al. [107] developed a system that detects hypovigilance, caused by drowsiness and inattention, using ECG and EMG signals. Inattention was controlled through a series of questions asked to the driver, through messaging or phone calls. On the other hand, drowsiness was controlled by allowing the subjects to drive continuously for 2 h using a simulator game in a controlled laboratory environment. The ECG and EMG data were recorded through disposable Ag–AgCl electrodes. The gathered physiological signals from the experiments were first pre-processed, in order to remove the artifacts and noise. Then, multiple higher-order spectral features were extracted, including the bispectrum, which is the Fourier transform of the second-order moment. From the bispectrum, other features were extracted, such as the (1) sum of the logarithmic amplitudes of the bispectrum (H1), (2) sum of the logarithmic amplitudes of the diagonal elements in the bispectrum (H2), and (3) first-order spectral moment of the amplitudes of diagonal elements in the bispectrum (H3). Furthermore, to enhance the accuracy of the results, the data collected from the two signals were fused using principal component analysis. Next, the extracted features were trained and classified, using linear discriminant analysis, quadratic discriminant analysis, and KNN classifiers. Finally, the bispectral features showed an overall accuracy of 96.75% for the H3 feature from the ECG signal with the KNN classifier. Moreover, an accuracy of 92.31% for the H2 feature from the EMG signal with the linear discriminant analysis classifier was achieved. As for the fused features, the results showed a maximum accuracy of 97.06% using the KNN classifier.

  - Fatigue detection using noncontact EMG and ECG system

Fu and Wang [108] proposed a noncontact onboard fatigue detection system that analyzes the changes in the EMG and ECG signals during driving. Fast independent component analysis and digital filters are used to process these signals. Eight volunteers participated in this study, in order to collect data and train the system. The data were gathered using the noncontact data acquisition system, without direct contact with the driver’s skin. The system consisted of two conductive knit fabrics, sewn on the car cushion, that collected the data while the subject was sitting on them. The acquired data were pre-processed to extract the homogeneous signal parts. Then, feature selection was applied using the Kolmogorov–Smirnov Z test, which yields that the EMG peak factor (p < 0.001) and maximum cross-relation curve of ECG and EMG features showed an evident change when the drowsiness state started. To train this model, Mahalanobis distance, a measure of distance based on correlations between variables, was used to obtain discriminant criterion. The system’s final results showed an accuracy of 86%.

5. Drowsiness detection with a combination of various biological signals

  - An approach using EEG and ECG signals

Awais et a. [109] proposed a DDD method that combines ECG and EEG features to increase the detection performance. The authors measured the difference between drowsy and alert states, using a dataset from 22 participants in a simulator-based driving environment. During this study, a collection of features was extracted from both EEG and ECG signals. The features extracted from EEG signals included frequency domain absolute and relative powers, as well as time-domain statistical and complexity measures. On the other hand, the features extracted from the ECG signals included the HR and HRV features. After the feature extraction, a paired t-test was used to select significant features only. All features are then combined and fed to an SVM classifier. The results proved that combining the features obtained from both signals outperformed the features obtained from a single type of signal. It also showed that the use of combined EEG/ECG features allowed for reducing the number of electrodes needed. An accuracy of 80.90% was achieved when using a single EEG and ECG electrodes.

  - DDD using EEG, EOG, and ECG signals with fuzzy wavelet packet-based feature-extraction algorithm

Khushaba et al. [110] presented a feature extraction method for extracting the most relevant features to identify the driver drowsiness state. The proposed fuzzy mutual information-based wavelet packet transforms the feature extraction method, and it is aimed to optimize the amount of data, in relation to drowsiness, extracted from EEG, EOG, and ECG signals. These data were used to classify the driver state to one of the predefined drowsiness levels, which are alert (class-1), slightly drowsy (class-2), moderately drowsy (class-3), significantly drowsy (class-4), and extremely drowsy (class-5). The dataset came from 31 volunteers, who used a simulated driving test environment. The video data were rated and labeled using majority voting. Then, the new fuzzy mutual information-based wavelet packet transform method was used to extract the features, including EEG features from the temporal, frontal, and occipital channels, as well as the eyeblink rate, blood pressure, and heart rate. Next, these features were dimensionally reduced using spectral regression-based linear discriminant analysis [111] and kernel-based spectral regression [112] methods. After that, training was applied using four classifiers: linear discriminant analysis, linear SVM, kernel SVM, and KNN. The final results showed that the proposed method achieved an accuracy of 95% with spectral regression and 97% for kernel spectral regression across different classifiers. 

**Table 6:** Biological-based drowsiness detection systems.

Ref.	Biological Parameters	Sensors	Extracted Features	Classification Method	Description	Quality Metric	Dataset
[79]	Brain activity	Bluetooth-enabled EEG headband and a commercial smartwatch	Relative EEG power ratio (power percentages)	SVM-based posterior probabilistic model	A real-time system used an SVM-based posterior probabilistic model to detect and classify drowsiness into three levels.	Accuracy: Drowsy case: 91.92% Alert case: 91.25% Warning case: 83.78%	Prepared their own dataset
[80]	Brain activity	EEG (silver surface electrode)	IMF of the EEG signal	ANN	Detection was based on the extraction of the IMFs from the EEG signal by applying the EMD method.	Accuracy: 88.2%	Prepared their own dataset
[81]	EEG signals and EEG spectrogram images	EEG Sensors	Energy distribution and zero-crossing distribution of the raw EEG signals, in-depth features of the EEG spectrogram, etc.	LSTM network	EEG-based drowsiness detection method. It used pre-trained AlexNet and VGG16 models to extract in-depth features from the EEG spectrogram images.	Accuracy: 94.31%	MIT/BIH polysomnographic EEG database [82]
[83]	EEG	EEG Sensors	The first quartile, median, range, and energy of the Hermite coefficients	ELM decision tree, KNN, least squares SVM, ANN, and naive Bayes	Detection was based on an adaptive Hermite decomposition for EEG signals. The Hermite functions were employed as basic functions.	Accuracy: ELM: 92.28% Sensitivity: ELM: 95.45%	MIT/BIH polysomnographic database [82]
[85]	EEG	Standard wet-electrode EEG and a cap-type dry-electrodeEEG	Multi-taper power spectral density	Extreme gradient boosting classifier	A framework for detecting instantaneous drowsiness with a 2-s length of EEG signal. It was implemented on a wireless and wired EEG to show its applicability in a mobile environment.	Accuracy: Wired EEG: 78.51% Wireless EEG: 77.22%. Sensitivity: Wired EEG: 78.5%, Wireless EEG: 68.3%	Prepared their own dataset
[87]	EEG	EEG sensors	F1–F9, extracted from Higuchi fractal dimension, complexity, and mobility characteristics of the original EEG signal, as well as all the EEG sub-bands	Extra trees classifier	Employed wavelet packet transform to extract the time domain features from a single-channel EEG signal. Eleven classifiers were tested in this work. The extra trees classifier had the best results.	Accuracy, sensitivity, and precision: Dataset1: 94.45%, 95.82%, and 96.14% Dataset2: 85.3%, 79.55%, and 90.02%	Dataset1: Fpz-Cz channel dataset [89,90] Dataset2:SVDD dataset [91]
[96]	EEG	EEG Sensors	Tsallis entropy, Renyi entropy, permutation entropy, log energy entropy, and Shannon entropy	Ensemble boosted tree classifier	Used AVMD to analyze and synthesize the EEG signals. By applying statistical analysis, five entropy-based features were selected. Ten classifiers were used, and the ensemble boosted tree classifier achieved the highest accuracy.	Accuracy: 97.19% Sensitivity: 97.01% Precision: 98.18%	MIT/BIH polysomnographic dataset [82]
[100]	Heart rate and blood volume changes	ECG and PPG	Features obtained from Bin-RP, Cont-RP, and ReLU-RP patterns	CNN	Used wearable ECG/PPG sensors to track the different patterns in HRV signals in a simulation environment and used CNN.	Best accuracy, sensitivity, and precision: ECG: 70%, 85%, and 71% PPG: 64%, 78%, and 71%	Prepared their own dataset
[101]	Heart rate	PPG	Frequency measurements (HF, LF, and HF/LF) extracted from PPG signals	Differentiating between two (HF, LF, and HF/LF) patterns	Detection is done by analyzing the changes in PPG signals frequency measurements (HF, LF, and HF/LF) that are obtained from measurements on fingers and earlobes	Accuracy: 8/9 = 88.8%	Prepared their own dataset
[102]	Heart rate	Wrist-worn wearable sensor and ECG sensor	HRV and activity of the autonomic nervous systems	Random Tree, RF, KNN, SVM, Decision Stump, etc.	Detection was based on the physiological data extracted from a wrist-worn wearable sensor and ECG sensor. Multiple ML algorithms for binary classification were used	The highest accuracy was more than 92% for the KNN algorithm	Prepared their own dataset
[103]	HRV	ECG electrodes	MeanNN, SDNN, RMSSD, TP, NN50, LF, HF, and LF/HF	Multivariate statistical process control	Detection was based on HRV analysis. Eight HRV features were monitored to detect the changes in HRV using the multivariate statistical process control anomaly detection method. The algorithm was validated by comparing its results with EEG-based sleep scoring.	Accuracy: 92%	Prepared their own dataset
[104]	Respiration	Three respiratory inductive plethysmography sensors	RRV and quality of the respiratory signals	Thoracic effort-derived drowsiness	An algorithm for DDD, based on the respiratory signal variations. It combined the analysis of the RRV and the quality level of the respiratory signals to detect the changes in the driver’s alertness status.	Sensitivity: 90.3%	Prepared their own dataset
[107]	ECG and EMG	Disposable Ag–AgCl electrodes	Features extracted from the bispectrum of the signals H1, H2, and H3	Linear discriminant analysis, quadratic discriminant analysis, and KNN classifiers	Detects hypovigilance caused by drowsiness and inattention using ECG and EMG signals. The gathered physiological signals from the experiments were first pre-processed. Then, multiple higher-order spectral features were extracted to be classified.	Accuracy and Sensitivity: ECG with KNN: 96.75% and 98% EMG with linear discriminant analysis: 92.31% and 96% Fused features with KNN: 97.06%	Prepared their own dataset
[108]	ECG and EMG	Two pieces of conductive knit fabric	EMG peak factor and maximum of the cross-relation curve of ECG and EMG	Discriminant criterion using Mahalanobis distance	A noncontact onboard DDD system studied the EMG and ECG signals changes during driving. Feature selection was applied using the Kolmogorov–Smirnov Z test.	Accuracy: 86%. Sensitivity: 91.38% Precision: 83.45%	Prepared their own dataset
[109]	ECG and EEG	Enobio-20 channel device	EEG signals time-domain statistical descriptors, complexity measures, power spectral measures, ECG signals HR and HRV’s LF, HF, and LF/HF ratio	SVM	Combined ECG and EEG features to detect drowsiness. After the feature extraction, a paired t-test was only used to select the significant features.	Accuracy: 80.9%	Prepared their own dataset
[110]	EEG, EOG, ECG	EEG, ECG, and EOG electrodes	EEG features from the temporal, frontal, and occipital channels EOG features: eyeblink rate ECG feature: blood pressure and heart rate	Linear discriminant analysis, linear SVM, kernel SVM, and KNN	The fuzzy mutual information-based wavelet packet transform method extracted the features. The features were dimensionally reduced, using spectral regression and kernel-based spectral regression methods. After that, four classifiers were applied.	Accuracy: Spectral regression: 95% Kernel spectral regression: 97%	Prepared their own dataset

Table 6 reveals that biological-based systems have reported accuracies between 70% and 97.19%, with [96] showing the highest accuracy. Most of them rely on brain activity signals for detection. These systems may be intrusive or invasive, depending on the utilized sensors. Further details are discussed later in Section 4. As mentioned earlier, these systems can detect drowsiness at an early stage.

### 3.3. Vehicle-Based Measures

This method depends on tracing and analyzing driving patterns. Every driver forms a unique driving pattern. Thus, the driving patterns of a drowsy driver can be easily distinguished from those of an alert driver. According to Pratama et al. [8], vehicular-based measures are the least investigated methods, due to the difficulty of precisely determining drowsy driving state features. Thus, many researchers combine this measure with image-based or biological measures [24,113,114]. The two most common detected vehicle-based measures, used to identify driver drowsiness, are steering wheel angle (SWA) and lane departure. Table 7 provides a list of DDD systems based on vehicle measures.

As for the SWA, it can be measured using angle sensors that are connected to the steering wheel. However, the way the data are collected may differ from one method to another. Lane departure feature can be acquired by tracking lane curvature, position, or curvature derivative. Below, we present some examples of vehicle-based DDD systems that use these measures.

1. Tracking drowsiness using SWA

McDonald et al. [113] proposed analyzing lane departure using SWA data and the RF algorithm. The authors compared their approach to another image-based drowsiness measure that used PERCLOS. The comparison showed that the SWA measure had higher accuracy, which reached 79% and could detect drowsiness 6 s in advance. At the same time, the PERCLOS method achieved 55% accuracy only. The algorithm was tested using a dataset (72 participants) from a study at the University of Iowa’s National Advanced Driving Simulator [115]. The modified observer rating of drowsiness scale extracted the drowsiness related to lane departure from raw simulator data. The readings were taken every one minute after departing out of the lane. As for the PERCLOS measure, the features were extracted from a video and captured using an eye detecting FaceLab software. Furthermore, the RF algorithm was trained by a series of decision trees, with a randomly selected feature.

2. Lateral distance using wavelet transform and neural network

Ma et al. [114] proposed a model that detects driver drowsiness based on lateral distance. The lateral distance can be acquired from fusing lane curvature, position, and curvature derivative. Those three raw features were obtained using the transportable instrumentation package system [116], with a video camera placed on the car’s front bumper. Moreover, this system uses real-time video recording to collect the driver’s facial and head movements data. The driver’s visual data were used as ground truth for the car’s data. The recorded car data was fed to TRW’s simulator, in order to extract lane-related signals in the frequency and time domain. After that, the signals were analyzed, along with the acquired footage of the driver’s face. Then, the data were fed to SVM and neural network algorithms for classification. As for the experimental results, all classification methods showed a detection accuracy higher than 90%.

3. Entropy features from SWA time series

This system uses SWA data to apply online fatigue detection. The data were collected from a sensor settled on the steering lever for 14.68 h and under real driving conditions. Li et al. [117] proposed a system that uses a fixed sliding window to extract the approximate entropy features from a SWAs time series data. Then, the approximate entropy features series are linearized, using an adaptive piecewise linear fitting, with a specific deviation. Then, the system calculates the warping distance between the linear features series to determine the driver’s alertness state. Finally, the alertness state, either “drowsy” or “awake,” is determined using a specially designed binary decision classifier. The system’s experimental results showed an accuracy of 84.85% for true detections of the “drowsy state” and 78.01% for the “awake” state’s true detections.

4. ANFIS based steering wheel feature selection

Arefnezhad et al. [118] presented a non-invasive DDD system based on steering wheel data. The system aimed to increase classification accuracy using feature selection strategies. The proposed selection method used adaptive neuro-fuzzy inference systems (ANFIS), a combination of filters and wrapper feature selection algorithms. The study was conducted in a simulated driving environment involving 39 bus drivers, resulting in a new dataset. Thirty-six features were extracted from the steering wheel data. These features were applied to four different filter indices. The output of each filter was fed to the fuzzy system to select the most important features. Then, an SVM classifier is used to classify the selected features and specify the drivers’ state. Finally, using a particle swarm optimization method, the classifier’s accuracy is used to optimize the parameters of the ANFIS. The final results showed an accuracy of 98.12%.

5. DDD based on steering wheel status

Chai et al. [119] presented a study on drowsiness monitoring, using data relating to steering wheel status. They used a driving simulator to collect 11 parameters for the steering wheel. Based on the correlation level with driver’s status, four parameters were selected:

- SW_Range_2: steering wheel angular velocity percentage in the range of 2.5–5°/s.
- Amp_D2_Theta: the area between the SWA, θ; the mean of θ is multiplied by the time the SWA is on the same side of the mean of θ.
- PNS: proportion of the time that the steering wheel remains stationary (±0.1°).
- NMRHOLD: number of times the steering wheel is held steady (within a certain threshold angle) for longer than 0.04 s. Steady means that the change in angle is lower than ±0.5°.

Three models were then built for drowsiness detection based on these parameters: a multilevel ordered logit (MOL) [120], SVM, and back propagation neural network (BPNN) model. Under the same classification conditions, the results showed that the MOL model had achieved an accuracy of 72.92%, much higher than the others. Thus, the authors concluded that using these four parameters, while considering the differences of individuals, the MOL model has outperformed the other two models.

**Table 7:** Vehicle-based drowsiness detection systems.

Ref.	Vehicle Parameters	Extracted Features	Classification Method	Description	Quality Metric	Dataset
[113]	Steering wheel	SWA	RF	Used SWA as input data and compared it with PERCLOS. The RF algorithm was trained by a series of decision trees, with a randomly selected feature.	Accuracy: RF- steering model: 79% PERCLOS: 55%	Prepared their own dataset
[114]	Lateral distance	Statistical features, derived from the time and wavelet domains, relevant to the lateral distance and lane trajectory	SVM and neural network	Detection was based on lateral distance. Additionally, it collects data of the driver’s facial and head movements to be used as ground truth for the vehicle data.	Accuracy: Over 90%	Prepared their own dataset
[117]	Steering wheel	SWA	Specially designed binary decision classifier	Used SWA data to apply online fatigue detection. The alertness state is determined using a specially designed classifier.	Accuracy: Drowsy: 84.85% Awake: 78.01%	Prepared their own dataset
[118]	Steering wheel	SWA, steering wheel velocity	ANFIS for feature selection, PSO for optimizing the ANFIS parameters, and SVM for classification	Detection was based on steering wheel data. The system used a selection method that utilized ANFIS.	Accuracy: 98.12%	Prepared their own dataset
[119]	Steering wheel	SW_Range_2, Amp_D2_Theta, PNS, and NMRHOLD	MOL, SVM, and BPNN	Used steering wheel status data. Using variance analysis, four parameters were selected, based on the correlation level with the driver’s status. MOL model performed best.	Accuracy: MOL: 72.92% SVM: 63.86% BPNN: 62.10%	Prepared their own dataset

Table 7 reveals that vehicle-based systems have reported accuracies between 62.1% and 98.12%, with [118] showing the highest accuracy. Most of them rely on the SWA feature. Generally, these systems are non-intrusive and -invasive.

### 3.4. Hybrid-Based Measures

A hybrid DDD system employs a combination of image-, biological-, and vehicle-based measures to extract drowsiness features, with the aim of producing a more robust, accurate, and reliable DDD system. This subsection presents some of the recently proposed hybrid DDD systems. Table 8 shows a list of those systems.

1. Driver assistance system, based on image- and vehicle-based features

Saito et al. [24] presented a driver assistance system with a dual control scheme. This system effectively identifies driver drowsiness based on eyelid’s state, steering wheel, and lane departure and takes control of the car, if needed. The assistance system initiates a partial control of the vehicle, in the case of a lane departure. The system gives the driver a chance to control the car and center it in the lane. If the driver does not take control of the vehicle within a specific time duration, the system assumes that the driver is unable to drive or is asleep. Thus, the system will take control and park the car. Twenty participants took part in this study. The data were mainly collected when the proposed assistance system was active. The driver status was determined through a series of mathematical operations and specified schemes from the study hypothesis. The study results showed accuracies of up to 100% in taking control of the car when the specified driving conditions were met.

2. Biomedical and motion sensors

Leng et al. [28] proposed a wearable device that uses motion and biomedical sensors to detect drowsiness using a mobile application. The system combines both drivers’ biosignals and vehicle measures to get an accurate result. It uses a self-designed wristband to detect biosignals and motion sensors to detect steering wheel movement. The wristband contains two main components: galvanic skin response and photoplethysmogram sensors that detect PPG signals. Additionally, a smartwatch’s built-in accelerometer and gyroscope sensors are used to detect the steering wheel’s linear acceleration and radian speed. Thus, the system starts by collecting data from the sensors. Then, the collected data are passed to the smartwatch, where they are processed and analyzed. After that, five features are extracted from the received biological raw data: heart rate, stress level, respiratory rate, adjustment counter, and pulse rate variability. Next, these five features, along with the steering wheel data, are fed to an SVM algorithm to detect the driver’s drowsiness state. After getting the classification result, the smartwatch alerts the driver, through a visual and vibration alarm. The system resulted in an accuracy of 98.3%.

3. Yawning, blinking, and blood volume pulse-based method

Yawning, blinking, and heart rate change can provide clues about the driver’s mental state. Based on that fact, Zhang et al. [121] proposed a DDD system that uses a smartphone’s camera as a non-contact optical sensor. The system captures image sequences and uses them as raw data to extract blink and yawn signals. Additionally, the extended-PPG signals, obtained from the image sequence, enable extracting blood volume pulse, without direct contact with the person under consideration. Using a multichannel second-order blind identification, the blood volume pulse, yawning, and blinking signals are simultaneously extracted from smartphone videos. The combined signals are then analyzed to estimate the blinking duration and frequency, HRV, and yawning frequency. Should any of the estimated parameters show a specific value, then drowsiness will be declared, and an alarm will sound from the phone. The system showed different sensitivity values, ranging up to 94%.

4. EEG signals’ spectral, head movement, and blink analysis

Mehreen et al. [29] extracted the drivers’ behavioral and biological features, using a lightweight, non-invasive, wearable headband to replace cameras and intrusive sensors in DDD systems. The proposed DDD system uses a combination of signals, acquired from a headband equipped with an accelerometer, gyroscope, and EEG electrodes. The dataset was collected in both drowsy and fresh state conditions, with the help of 50 volunteers, using a driving simulator. To increase the robustness and acquire better accuracy results, the authors combined the features extracted from the head movement analysis, eye blinking, and spectral signals to make a feature vector. The backward feature selection method was then applied on the feature vector over various classifiers. When fed with the whole feature vector, the linear SVM performed the best, with an accuracy of 86.5% before feature selection and 92% after feature selection was applied.

5. DDD using image-, biological-, and vehicle-based features fusion

De Naurois et al. [122] investigated the possibility of predicting when a drowsiness level is reached by utilizing the same information used to determine drowsiness. Furthermore, they explored whether including additional data, such as the participant information and driving time, would improve the detection and prediction accuracy. Using a car simulator, 21 participants drove for 110 min, under special conditions that induced drowsiness. The researchers measured biological, behavioral, and vehicle drowsiness features. Such features include heart rate and variability, respiration rate, blink duration, frequency, PERCLOS, head and eyelid movements, time-to-lane-crossing, position on the lane, speed, and SWA. Two models that use ANN were developed, one for detecting the drowsiness degree, and the other is for predicting the time needed to reach a specific drowsiness level. Both models ran every minute. Different combinations of the features were tested during this study. Finally, the models showed that it could detect drowsiness levels (with a mean square error of 0.22) and predict the time to reach a specified drowsiness level (with a mean square error of 4.18 min).

6. Combined EEG/NIRS DDD system

In their study, Nguyen et al. [123] introduced an approach that combines EEG and near-infrared spectroscopy (NIRS) to detect driver drowsiness. NIRS is a spectroscopic method that utilizes the electromagnetic spectrum near-infrared region. Multiple biological signals were recorded during a simulated driving task over nine subjects. These measurements included the neuronal electrical activity (using EEG signals), tissue oxygenation (using NIRS), eye movement (using EOG signals), and heart rate using (ECG signals). The features studied to determine the drowsiness state included heart rate, alpha and beta bands power, blinking rate, and eye closure duration. Statistical tests showed that the frontal beta band and oxygenation change showed the most significant difference between the alert and drowsy states and thus were chosen as the most relevant parameters from the EEG and NIRS signals for the study. Fisher’s linear discriminant analysis method was used for driver’s state classification. In addition, the time series analysis was employed to predict drowsiness. Although multimodal data were collected, only EEG and NIRS were used for further analysis because the other data did not clearly correspond to the drivers’ alertness state changes. The proposed system resulted in an accuracy of 79.2%.

7. DDD using EEG, EOG, and contextual information

In this study, Barua et al. [124] proposed an automatic sleepiness detection scheme using EOG, EEG, and contextual information. The features extracted from the EEG signal power spectra included five frequency characteristics and four power ratios. Blinking duration and PERCLOS were calculated from the EOG signal. The contextual information features used in the proposed system included driving conditions, such as the lighting condition and driving environment, along with the sleep/wake predictor value. Three feature selection algorithms were employed to select the most suitable feature combination from the abovementioned features pools. These algorithms were the univariate feature selection method, sequential forward floating selection wrapper method, and minimum redundancy maximum relevance method. KNN, SVM, case-based reasoning, and RF were used for classification. The authors considered two classification cases: multiclass classification (alert, somewhat sleepy, or sleepy) and binary classification (alert or sleepy). 

The study used data from 30 drivers, who used a driving simulator. Overall, the SVM classifier showed the best performance, with 79% accuracy for the multiclass classification and 93% for binary classification. The study clearly showed that adding contextual information to the signals data boosted the classification accuracy by 4% and 5% for multiclass and binary classification, respectively.

8. DDD with a smartphone

Dasgupta et al. [125] proposed a DDD and warning system using a smartphone. This proposed system was one of the first attempts to combine voice cues with PERCLOS to detect drowsiness. Thus, they have prepared their own dataset, called the Invedrifac dataset [126]. The presented work uses three verification stages in the process of detection. The first stage computes the PERCLOS feature, obtained from a smartphone’s front camera images. If the PERCLOS value crosses a certain threshold, the system initiates the second stage by requesting the driver say his full name. Having been labeled a drowsy driver in the first two stages, the system asks the driver to tap the smartphone screen within 10 s. If the condition is not met, drowsiness is verified, and an alarm is initiated. The proposed framework used a linear SVM classifier and resulted in a final accuracy of 93.33%.

9. DDD using ensemble ML and hybrid sensing

In [127], Gwak et al. investigated the feasibility of detecting early drowsiness based on hybrid measures, namely vehicle-based, physiological, and behavioral signs, to implement a detection system. Sixteen participants were involved in this study. A total of 80 features were extracted from the measured data and videos. The study consisted of three main parts. In the first part, the drivers’ physiological signals, driving performance, and behavioral drowsiness signs were recorded, using a driving simulator and monitoring system. Then, classification was performed using two different classification methods: RF classifier and majority voting, using logistic regression, SVM, and KNN. In the case of majority voting, sequential backward feature selection was performed, and then classification was applied. On the other hand, in the case of RF, the number of estimators and features used was optimized to get better classification performance. Finally, the performance of the algorithms was evaluated. This study, which followed Zilberg’s criteria [128] in labeling the drowsiness levels, has focused on differentiating between alert and slightly drowsy and alert and moderately drowsy states. The results showed that the RF classifier gave the best results, with 82.4% accuracy of alert vs slightly drowsy case. In contrast, majority voting performed the best for alert vs moderately drowsy case, at an accuracy of 95.4%.

**Table 8:** Hybrid-based drowsiness detection systems.

Ref.	Sensors	Hybrid Parameters	Extracted Features	Classification Method	Description	Quality Metric	Dataset
[24]	Automatic gearbox, image-generating computers, and control-loaded steering system	Image- and vehicle-based features	Latera position, yaw angle, speed, steering angle, driver’s input torque, eyelid opening degree, etc.	A series of mathematical operations, specified schemes from the study hypothesis	A system that assists the driver in case drowsiness is detected to prevent lane departure. It gives the driver a specific duration of time to control the car. If not, the system controls the vehicle and parks it.	Accuracies up to 100% in taking control of the car when the specified driving conditions were met	Prepared their own dataset
[28]	PPG, sensor, accelerometer, and gyroscope	Biological- and vehicle-based features	Heart rate, stress level, respiratory rate, adjustment counter, and pulse rate variability, steering wheel’s linear acceleration, and radian speed	SVM	It collected data from the sensors. Then, the features were extracted and fed to the SVM algorithm. If determined drowsy, the driver is alerted via the watch’s alarm.	Accuracy: 98.3%	Prepared their own dataset
[121]	Smartphone camera	Biological- and image-based features	Blood volume pulse, blinking duration and frequency, HRV, and yawning frequency	If any of the detected parameters showed a specific change/value	Used a multichannel second-order blind identification based on the extended-PPG in a smartphone to extract blood volume pulse, yawning, and blinking signals.	Sensitivity: Up to 94%	Prepared their own dataset
[29]	Headband, equipped with EEG electrodes, accelerometer, and gyroscope	Biological- and behavioral-based features	Eyeblink patterns analysis, head movement angle, and magnitude, and spectral power analysis	Backward feature selection method applied followed by various classifiers	Used a non-invasive and wearable headband that contains three sensors. This system combines the features extracted from the head movement analysis, eye blinking, and spectral signals. The features are then fed to a feature selection block followed by various classification methods. Linear SVM performed the best.	Accuracy, sensitivity, and precision: Linear SVM: 86.5%, 88%, and 84.6% Linear SVM after feature selection: 92%, 88%, and 95.6%	Prepared their own dataset
[122]	SCANeR Studio, faceLAB, electrocardiogram, PPG sensor, electro-dermal activity, Biopac MP150 system, and AcqKnowledge software	Biological-, image-, and vehicle-based features	Heart rate and variability, respiration rate, blink duration, frequency, PERCLOS, head and eyelid movements, time-to-lane-crossing, position on the lane, speed, and SWA	ANN	Included two models that used ANN. One is for detecting the drowsiness degree, and the other is for predicting the time needed to reach a specific drowsiness level. Different combinations of the features were tested.	Overall mean square error of 0.22 for predicting various drowsiness levels Overall mean square error of 4.18 min for predicting when a specific drowsiness level will be reached	Prepared their own dataset
[123]	EEG, EOG, ECG electrodes, and channels	Biological-based features and NIRS	Heart rate, alpha and beta bands power, blinking rate, and eye closure duration	Fisher’s linear discriminant analysis method	A new approach that combined EEG and NIRS to detect driver drowsiness. The most informative parameters were the frontal beta band and the oxygenation. As for classification, Fisher’s linear discriminant analysis method was used. Additionally, time series analysis was employed to predict drowsiness.	Accuracy: 79.2%	MIT/BIH polysomnographic database [82]
[124]	Multi-channel amplifier with active electrodes, projection screen, and touch screen	Biological-based features and contextual information	EEG signal: power spectra, five frequency characteristics, along with four power ratiosEOG signal: blinking duration and PERCLOS contextual information: the driving conditions (lighting condition and driving environment) and sleep/wake predictor value.	KNN, SVM, case-based reasoning, and RF	Used EOG, EEG, and contextual information. The scheme contained five sub-modules. Overall, the SVM classifier showed the best performance.	Accuracy: SVM multiclass classification: 79% SVM binary classification: 93% Sensitivity: SVM multiclass classification: 74% SVM binary classification: 94%.	Prepared their own data
[125]	Smartphone	Image-based features, as well as voice and touch information	PERCLOS, vocal data, touch response data	Linear SVM	Utilized a smartphone for DDD. The system used three verification stages in the process of detection. If drowsiness is verified, an alarm will be initiated.	Accuracy: 93.33%	Prepared their own dataset called ‘Invedrifac’ [126]
[127]	Driving simulator and monitoring system	Biological-, image-, and vehicle-based features	80 features were extracted: PERCLOS, SWA, LF/HF, etc.	RF and majority voting (logistic regression, SVM, KNN) classifiers	Vehicle-based, physiological, and behavioral signs were used in this system. Two ways for labeling the driver’s drowsiness state were used, slightly drowsy and moderately drowsy.	Accuracy, sensitivity, and precision: RF classifier: Slightly drowsy labeling: 82.4%, 84.1%, and 81.6% Majority voting: Moderately drowsy labeling: 95.4%, 92.9%, and 97.1%	Prepared their own dataset

Table 8 reveals that hybrid-based systems have reported accuracies between 79% and 99%, with [24] showing the highest accuracy. Most of them rely on at least one biological feature for detection. This type of system may be intrusive or invasive, depending on the features they use. Hybrid-based systems’ advantages and challenges rely on the combination of features they utilize to detect drowsiness. Further details are discussed in the following section. 

## 4. Challenges

Since the launching of Volvo’s first DDD system in 2007 [129], the technology has evolved tremendously. However, there are still many challenges and issues that face researchers. In this section, we discuss the challenges in detecting drivers’ drowsiness.

Most researchers generally apply their studies in a virtual or simulated environment and build their final system results based on the simulation output. However, those results do not necessarily represent real-life driving situations. Moreover, in such simulated environments, the study is narrowed by specific drowsiness scenarios, eliminating the vast range of possibilities and conditions a driver faces in real-life scenarios, which, in return, affects the system reported accuracy [17,33,59,63,68,121,123,127]. Such issues may be overcome by validating the system’s results through equivalent testing in real-life driving sessions.

The major challenge of image-based DDD systems is the struggle to track and recognize high-quality head and facial data. This challenge is due to the dependence on the efficiency and quality of the used equipment, as well as the driver and environmental conditions. Other issues associated with such systems are the presence of additional features on the face, such as sunglasses, a beard, or a mustache, that may cover the eye or mouth and lead to a system failure. Additional challenges include the random head movement [38,41,52], different skin colors, various lighting conditions [55,130], face’s distance from the camera, different face structure based on race, and real-time video analysis that require powerful computing resources [103]. All of that may reduce the accuracy or even lead to false detection.

As for the biological-based systems, the studies show that such systems are the most accurate in detecting early drowsiness signs. However, the main issue with such systems is the equipment and sensors associated with them. Such equipment types are not comfortable, in some cases, as they must be attached to the driver’s body during the journey. Additionally, the used biological sensors are vulnerable to slight movement, which may produce some noise in the extracted signals, reducing the accuracy [100]. 

Another challenge that may specifically affect biological- and hybrid-based systems is the hardware complexity and limitations. For example, in [85], the system faced technical challenges, including hardware and compatibility issues. The wireless EEG hardware could not collect as much data as the wired EEG did, partly because of the instability of the used dry electrodes. Additionally, the EOG sensor R100 that was used in the experiment had compatibility issues with the used wireless EEG system. They could not be used simultaneously because of interference. Moreover, in [124], Barua et al. pointed out that measuring EEG signals alone in a real driving scenario could lead to features that are not reliable enough to produce high accuracy. They added that more complex sensors need to be employed for better system performance, in order to provide multimodal information, i.e., additional features that can enhance accurate sleepiness detection.

In comparison, three significant challenges face vehicle-based DDD systems. First, weather conditions. If a driver is driving in harsh weather, with strong wind or rain, the car will naturally deviate out of the lane, leading the system to generate false results. Another challenge is the geometric conditions of roads. In some cases, the driver may drive on a steep and bumpy road, causing the car to vibrate, divert, and the steering wheel to shake, causing the collected data to become unreliable. Furthermore, some vehicle-based systems do not extract drowsiness signs precisely, leading to inaccurate detection results. Thus, many systems use an additional measure with vehicle measures, in oder to increase the system’s accuracy. 

Nevertheless, many future research directions address these issues to enhance the accuracy of these systems. For example, recent research [121] uses some lightweight sensors or analyzes biological signals based on video data, which does not require direct contact with the driver and maintains a high accuracy rate at the same time. Such an approach eases the detection process and shows a promising future for such systems. Besides, more and more systems are designed with hybrid measures that give not only higher accuracy but also each separate measure in such systems complement each other, which increases the system’s efficiency.

Inspired by [131], we attempt in this work to quantitatively describe the challenges facing DDD systems. The challenges are summarized and listed in Table 9, against the three principal DDD systems. Each challenge in the table is labeled as low, medium, high, or not applicable (N/A), in order to describe the significance level of that challenge on the performance of each DDD system type. Hybrid DDD systems are a combination of the other three systems. Thus, they were not included in the table, since the listed challenges may impact them in various degrees.

Table 9 shows that the challenges facing the image-based systems are mainly related to the face region, which is the region of interest, where most features are extracted. On the other hand, the biological-based system’s challenges relate to the used equipment and hardware setup. In contrast, vehicle-based systems have fewer challenges, with the main one being the inability to extract drowsiness signs precisely. Therefore, using vehicle-based techniques with other measures would result in a more reliable hybrid system.

## 5. Discussion

A thorough literature review showed that various methods implement drowsiness detection and terminate possible hazards while driving. Moreover, the technological development and continuous advancement in the artificial intelligence domain solved many challenges faced by such systems and enhanced their performance. This section compares the DDD systems, in terms of their practicality and reliability, as reported in the literature, and discusses the four drowsiness detection measures mentioned previously. System practicality describes the system’s effectiveness, in terms of invasiveness, intrusiveness, cost, ease of use, and accuracy, in detecting true drowsiness states. Intrusive systems stand for systems that use sensors with probes that are attached to the surface of the human body. In contrast, invasive systems refer to systems that use invasive sensors, where the probe must enter the human body and come into contact with bodily fluid. Table 10 summarizes the practical characteristics of each of the four types of DDD systems.

The first type, image-based systems, are generally considered practical because they are non-intrusive, non-invasive systems, as well as cost-efficient and automatic, in the sense that there is no need to set up any sensor each time the system is used. Such systems use various types of cameras, such as webcams [39,49,54], smartphone cameras [121,125], or thermal cameras [16,38]. The cameras are set at a specific distance from the driver to collect data without obstructing the driver’s view. In terms of detection accuracy, the DDD image-based systems differ in their results. Since they monitor features that are highly correlated to drowsiness, such as yawning, blinking, head movement, and eye closure, most of them have achieved high accuracy, between 85% to 99%, as shown in systems [17,52,54,55,59]. However, it should be noted that such systems are affected by multiple factors, as mentioned previously in the challenges section, and are often implemented and tested in a controlled environment or using existing DDD video datasets [30,33,49,59,63,68]. Thus, the drowsiness signs are mostly simulated, where the driver is asked to mimic specific signs during the data collection part. Hence, all of these factors reduce the reliability of the image-based systems.

The second type is biological-based DDD systems. The practicality characteristics of such systems are based on the sensors used to detect the targeted biological feature. Such sensors are usually required to be set up every time they are used, and if they were high-quality, they would be costly. In terms of intrusiveness and invasiveness, various types of sensors are used in such systems, and these characteristics differ, based on the used one. Take, for example, systems [85,103], where they require the driver to put electrodes on the scalp before driving. In this case, these systems are considered invasive and intrusive, making them impractical because it is burdensome for drivers to keep them attached while driving. In comparison, systems [79,100] have used devices like a headband with sensors or a smartwatch, which are biological devices that can easily be worn and attached to the driver’s head and arm for data collection. Those devices are non-invasive and intrusive and do not disturb or obstruct the driver. Nonetheless, other biological-based systems were designed more comfortably and did not require the driver to put them on before driving. Such systems use sensors attached to the seat or steering wheel, making them comfortable for long rides. Examples include systems [101,108]. 

In [101], a head support that contains PPG sensors to measure the pulse was connected to the simulator chair. While in [108], Fu and Wang used two pieces of conductive knit fabric that was sewed to the seat cushion. The fabric collected data, while the driver was seated. Regardless of the hardware or method implemented for drowsiness detection, the accuracy of the biological-based systems tends to be high. These systems trace the human body’s biological signals that reflect the very initial changes in the human alertness state, which allows them to make an accurate detection when the drowsiness signs appear. The accuracies of the reviewed biological-based systems were between 77–97%. Thus, unlike image-based systems, biological-based systems are considered highly reliable, when it comes to detecting real-time drowsiness.

In the third type, vehicle-based systems, these systems take their readings from vehicle-related parameters, as the name implies. Thus, they are also non-intrusive and -invasive systems. Furthermore, like image-based systems, they automatically start the detection process, without the need for initial preparations, but their cost differs, depending on the sensor used. In terms of the final accuracy, vehicle-based systems have scored the lowest accuracy, compared to other types of systems. For example, systems [117,119], in Table 7, have shown an accuracy of 78% and 62.1%, respectively. Nevertheless, such systems did not obstruct the driver in any way. However, this type of system is the least reliable for detecting drowsiness, due to the type of features they track, such as SWA and lane departure. Such features cannot solely detect drowsiness accurately.

In some studies, researchers used additional measures to enhance the effectiveness of vehicle-based systems. Such systems have resulted in the fourth type of DDD systems, which we referred to as hybrid-based systems. As mentioned before, hybrid systems have many combinations using vehicle-, image-, biological-, image-based measures, etc. These systems may be intrusive or invasive, easy to use, or costly, depending on the measures combined and hardware used. For example, in [29], the authors have used a non-invasive and wearable headband that contains three sensors to detect the EEG signals, head movement, and blinking. Thus, this system has reduced the detection surface, where the sensors can be placed into one headband. However, the head band must be worn at all the time during the ride, which may be uncomfortable for the driver. As shown in Table 10, most of the hybrid-based DDD systems have achieved accuracy exceeding 85%, which indicates the effectiveness and practicality of such systems.

Overall, according to the literature, image-based systems gave more than 90% accuracy within a simulated testing environment or using an existing dataset. This measure has proven reliable in detecting drowsiness, once visual signs start appearing. However, being implemented in a controlled environment is a major drawback, which argues the need to be tested in real-life driving scenarios, under highly safe measures, for it to be considered reliable. On the other hand, biological systems detect drowsiness early because they depend on internal drowsiness signs, which usually appear before visual signs [9]. Thus, they can give an early alarm that alerts the driver, significantly reducing the chances of falling asleep. 

In contrast, vehicle-based systems resulted in the least accuracy. Additionally, as this measure’s results are unreliable alone, such systems are usually accompanied by another to validate the results of the vehicle-based measures. The hybrid-based systems showed a high performance; however, their multiple drowsiness measures distinguished this type of system. Notably, the various combinations of the hybrid systems have increased their performance and accuracy; additionally, they overcame some of the limitations that each measure has individually. In conclusion, using an accurate drowsiness detection system is one of the essential factors in reducing drowsiness-related car accidents. Furthermore, as the hybrid systems showed that they are highly reliable, they are the best option for drowsiness detection. 

Our paper classifies and reviews the latest DDD systems. Each system presented in this paper is accompanied by a detailed exposition of the involved features, classification algorithms, and datasets used. Our review also compares the systems, in terms of cost, invasiveness, intrusiveness, ease of use, and classification quality metrics (accuracy, sensitivity, and precision). Furthermore, the paper discusses the current challenges in DDD literature and sheds light on future research directions.

## 6. Future Trends in Drowsiness Detection Systems

Mobile phones have been introduced in literature [68,132] as an inexpensive alternative to collect driving data. Nowadays, mobile phones are equipped with at least two cameras and multiple sensors. Additionally, they can connect with a wide range of sensors through Bluetooth or other wireless technologies. When attached to the driver’s dashboard, a mobile phone’s front camera can collect various visual parameters, including eye features, mouth features, and head movements. Furthermore, the rear camera is capable of detecting vehicle-based features, such as lane departure and change in orientation, among others. Most mobile phones are also equipped with GPS sensors, an accelerometer, a gyroscope, and a magnetometer, which also could describe the car’s direction and orientation, leading to a better understanding of the driving experience. The phone’s microphone can also be used to collect data about the driver. 

The possibility of connecting sensors to a mobile phone using various wireless technologies allows the use of various biological sensors to collect the driver’s data seamlessly. For example, ECG, EEG, EMG, or PPG sensors can be attached to the driver’s body or embedded within the seat or steering wheel for more convenience. 

The data collected by the phone are analyzed using pre-trained machine learning models to infer the driver’s drowsiness status. While the use of machine learning algorithms on a mobile phone is possible, the use of deep learning is challenging and could lead to delayed inference times. Therefore, it is proposed to equip new mobile phones with chips, optimized for artificial intelligence [68], that facilitate the use of deep learning [133] for drowsiness detection on mobile platforms in real-time. 

Cloud system architecture has also been used to collect multi-sensor data from smartphones about drivers to analyze their driving behaviors and study their drowsiness patterns [132]. Developers use the gathered data to produce applications that consider contextual driving situations and personalized driver traits. Despite their advantages, the inherent latency in cloud systems makes cloud-based applications not favorable for driver drowsiness detection systems, where real-time decisions must be made [134]. An alternative method, with low latency, is multi-access edge computing (MEC). The MEC technology brings computing power and storage resources to the edge of the mobile network, instead of the central cloud server approach. MEC has been used in various mobile applications, where it showed fewer delays than cloud systems. The use of MEC-based DDD systems over 5G networks would lead to real-time decisions, which, in turn, provides safety to the driver [134].

DDD systems not only assure the safety of the driver and companions but also other passengers on the road. When the DDD system detects that the driver is drowsy, it signals an alarm (such as a flickering light) to other vehicles on the road, warning them that the driver is drowsy and to take caution [135]. The car could also be a member of an Internet of vehicle network, an IoT network involving vehicles. In such a setting, vehicles send live data that includes the driver’s vital signals over a wireless medium, such as 5G [136]. The data are collected and analyzed in a traffic management platform, which, in the case of detected drowsiness, sends an alert signal to the driver to reduce the speed or park the car. It could also run an autopilot to take over the vehicle and park it safely. On the other hand, the platform can also contact neighboring vehicles in the networks to warn them about the drowsy driver.

A significant limitation in most proposed DDD systems is their dependence on limited datasets that were produced in simulated environments [4]. The accuracy of these systems could be increased by obtaining more data from various drivers in actual vehicles, where factors such as the ambient light, road surface vibrations, and individual differences among drivers are considered. This requires the use of deep learning, which can be done locally by equipping the vehicles with AI-enabled processors and GPUs [4].

## 7. Conclusions

Over the past decade, the drowsiness detection field has experienced significant enhancements, due to technological advancements in IoT, sensor miniaturization, and artificial intelligence. This paper has presented a detailed and up-to-date review of the driver drowsiness detection systems that have been implemented in the last ten years. It has described the four main approaches followed in designing DDD systems and categorized them based on the type of drowsiness indicative parameters employed. These four categories are image-, biological-, vehicle-, and hybrid-based systems. The paper has provided a detailed description of all the presented systems, in terms of the used features, implemented AI algorithms, and datasets used, as well as the resulting system accuracy, sensitivity, and precision. 

Furthermore, the review has highlighted the current challenges in the DDD field, discussed the practicality of each DDD system, and discussed the current trends and future directions that aim to utilize affordable, easy-to-use, and practical methods to improve accuracy and reliability. 

We expect 5G networks to play a prominent role in enhancing DDD systems. With 5G connectivity, future DDD systems will be based on real driving scenarios. The data will be obtained from various drivers in actual vehicles, where factors such as ambient light, road surface vibrations, and individual differences among drivers are considered. The use of 5G connectivity will also enable the use of multi-access edge computing power for deep learning, resulting in highly accurate real-time decisions. Vehicles are expected to operate as members of Internet of vehicle networks, enabling the network to warn the drowsy driver, take control of the car (if needed), and contact neighboring vehicles in the network to alert them about the weary driver. These technologies will lead to safer roads and pave the way towards realizing smart cities.

We conclude by emphasizing that DDD technology has enormous market potential. Many car manufacturers, such as Toyota and Nissan, have recently installed or upgraded driver assistance devices in their products. The artificial intelligence and deep learning fields are developing tremendously. Soon, the DDD systems will most likely evolve, enabling the formation of smart cities.

## Figures and Tables

**Table 1:** Karolinska sleepiness scale, adapted from [19].

Scale	Verbal Description
1	Extremely alert
2	Very alert
3	Alert
4	Fairly alert
5	Neither alert nor sleepy
6	Some signs of sleepiness
7	Sleepy, but no effort to keep alert
8	Sleepy, some effort to keep alert
9	Very sleepy, great effort to keep alert

**Table 2:** Wierwille and Ellsworth drowsiness scale.

Levels	Verbal Description
1	Not drowsy
2	Slightly drowsy
3	Moderately drowsy
4	Significantly drowsy
5	Extremely drowsy

**Table 3:** Some of the image-based measures.

Features	Description
Blink frequency [30]	The number of times an eye closes over a specific period of time.
Maximum closure duration of the eyes [30]	The maximum time the eye was closed. However, it can be risky to delay detecting an extended eye closure that indicates a drowsy driver.
Percentage of eyelid closure (PERCLOS) [31]	The percentage of time (per minute) in which the eye is 80% closed or more.
Eye aspect ratio (EAR) [32]	EAR reflects the eye’s openness degree. The EAR value drops down to zero when the eyes are closed. On the other hand, it remains approximately constant when the eye is open. Thus, the EAR detects the eye closure at that time.
Yawning frequency [33]	The number of times the mouth opens over a specific period of time.
Head pose [34]	Is a figure that describes the driver’s head movements. It is determined by counting the video segments that show a large deviation of three Euler angles of head poses from their regular positions. These three angles are nodding, shaking, and tilting.

**Table 5:** Some biological-based measures.

Biological Signals	Description
Electroencephalography (EEG) [73]	An EEG signal is a monitoring method that records the brain’s electrical activity from the scalp. It represents the microscopic activity of the brain’s surface layer underneath the scalp. Based on the frequency ranges (0.1 Hz–100 Hz), these signals are categorized as delta, theta, alpha, beta, and gamma.
Electrocardiography (ECG) [74]	ECG signals represent the electrical activity of the heart, which are acquired using electrodes placed on the skin. ECG monitors heart functionality, including heart rhythm and rate.
Photoplethysmography (PPG) [75]	PPG signals are used to detect blood volume changes. These signals are measured at the skin’s surface using a pulse oximeter. It is often used for heart rate monitoring.
Heart rate variability (HRV) [76]	HRV signals are used to monitor the changes in the cardiac cycle, including the heartbeats.
Electrooculography (EOG) [77]	EOG signals are used to measure the corneo-retinal standing potential between the front and back of the human eye and record the eye movements.
Electromyography (EMG) [78]	EMG signals are the collective electric signals produced from muscles movement.

**Table 9:** DDD systems challenges.

	System Type	Imaged-Based	Biological-Based	Vehicle-Based
Challenges				
Difficulty in extracting drowsiness signs, due to facial characteristics/skin color		High	N/A	N/A
Difficulty in extracting drowsiness signs, due to objects that cover the face		High	N/A	N/A
Driver’s posture and distance from the dashboard		High	Low	N/A
Real-time video analysis		Medium	N/A	N/A
Driver movement		High	High	N/A
Noisy sensor measurements		Low	High	Low
Monitoring equipment and sensors inconvenience		Low	Medium	Low
Influence of environmental conditions (weather/illumination)		High	Low	Medium
Influence of the road conditions and geometry		Low	Low	High
Hardware complexity and limitations		Low	High	Low
Drowsiness signs extraction precision		Low	Low	High
Testing under real (not simulated) driving conditions		Medium	Medium	Medium

**Table 10:** DDD systems comparison, based on practicality.

	Practicality Features	Intrusiveness	Invasiveness	Reported Accuracies in DDD Literature	Cost	Ease of Use
DDD Systems Types						
Image-based systems		Non-intrusive	Non-invasive	High accuracy, between 72.25–99.59%	Generally low-cost	Automatic—no required set up or user intervention
Biological-based systems		Depends on the hardware and method used	Depends on the hardware and method used	High accuracy, between 70–97.19%	Expensive when high-quality sensors are used	May require set up, user intervention, or wearing sensors
Vehicle-based systems		Non-intrusive	Non-invasive	Low accuracy, as low as 62.1%	Mostly comes as an expensive car accessory	Automatic—no required set up or user intervention
Hybrid-based systems		Depends on the hardware and method used	Depends on the hardware and method used	High accuracy, between 79–99%	Cost depends on the used hardware	May require set up, user intervention, or wearing sensors

## Abbreviations

The nomenclature abbreviations, shown in Nomenclature, were used in this manuscript.

Nomenclature	
*NHTSA*	National highway traffic safety administration
*DDD*	Driver drowsiness detection
*IoT*	Internet of things
*KSS*	Karolinska sleepiness scale
*ML*	Machine learning
*TP*	True positive
*TN*	True negative
*FP*	False positive
*FN*	False negative
*NTHUDDD*	National Tsuing Hua university drowsy driver detection
*PERCLOS*	Percentage of eyelid closure
*EAR*	Eye aspect ratio
*SVM*	Support vector machine
*KNN*	K-nearest neighbor
*SHRP2*	Strategic highway research program results
*RF*	Random forest
*ANN*	Artificial neural networks
*CNN*	Convolutional neural network
*FD-NN*	Fully designed neural network
*TL-VGG16*	Transfer learning in VGG16—VG16 is a 16-layers deep CNN architecture, named after the Visual Geometry Group from Oxford
*TL-VGG19*	Transfer learning in VGG19—VG19 is a 19-layers deep CNN architecture
*LSTM*	Long short-term memory
*RNN*	Recursive neural network
*ROI*	Region of interest
*EM-CNN*	Eye and mouth CNN
*EMD*	Empirical mode decomposition
*IMF*	Intrinsic mode functions
*EEG*	Electroencephalography
*ECG*	Electrocardiography
*PPG*	Photoplethysmography
*HRV*	Heart rate variability
*EOG*	Electrooculography
*EMG*	Electromyography
*ELM*	Extreme learning machine
*SVDD*	Simulated virtual driving driver
*AVMD*	Adaptive variational mode decomposition
*RPs*	Recurrence plots
*RRIs*	R–R intervals
*Bin-RP*	Binary recurrence plot
*Cont-RP*	Continuous recurrence plot
*ReLU-RP*	Thresholded recurrence plot
*ReLU*	Rectified linear unit
*HF*	High frequency
*LF*	Low frequency
*LF/HF*	Low to high frequency
*MeanNN*	Mean of RRI
*SDNN*	Standard deviation of RRI
*RMSSD*	Root means square of the difference of adjacent RRI
*TP*	Total power which is the variance of RRI
*NN50*	Number of pairs of adjacent RRI spaced by 50 ms or more
*RRV*	Respiratory rate variability.
*H1*	Sum of the logarithmic amplitudes of the bispectrum
*H2*	Sum of the logarithmic amplitudes of the diagonal elements in the bispectrum
*H3*	First-order spectral moment of the amplitudes of diagonal elements in the bispectrum
*SWA*	Steering wheel angle
*ANFIS*	Adaptive neuro-fuzzy inference systems
*MOL*	Multilevel ordered logit
*BPNN*	Back propagation neural network
*NIRS*	Near-infrared spectroscopy
*MEC*	Multi-access edge computing
